# Induction of OTUD1 by RNA viruses potently inhibits innate immune responses by promoting degradation of the MAVS/TRAF3/TRAF6 signalosome

**DOI:** 10.1371/journal.ppat.1007067

**Published:** 2018-05-07

**Authors:** Liting Zhang, Jin Liu, Liping Qian, Qian Feng, Xiaofang Wang, Yukang Yuan, Yibo Zuo, Qiao Cheng, Ying Miao, Tingting Guo, Xiaofeng Zheng, Hui Zheng

**Affiliations:** 1 Institutes of Biology and Medical Sciences, Soochow University, Suzhou, China; 2 Jiangsu Key Laboratory of Infection and Immunity, Soochow University, Suzhou, China; 3 Department of Biochemistry and Molecular Biology, School of Life Sciences, Peking University, Beijing, China; University of North Carolina at Chapel Hill, UNITED STATES

## Abstract

During RNA virus infection, the adaptor protein MAVS recruits TRAF3 and TRAF6 to form a signalosome, which is critical to induce the production of type I interferons (IFNs) and proinflammatory cytokines. While activation of the MAVS/TRAF3/TRAF6 signalosome is well studied, the negative regulation of the signalosome remains largely unknown. Here we report that RNA viruses specifically promote the deubiquitinase OTUD1 expression by NF-κB-dependent mechanisms at the early stage of viral infection. Furthermore, OTUD1 upregulates protein levels of intracellular Smurf1 by removing Smurf1 ubiquitination. Importantly, RNA virus infection promotes the binding of Smurf1 to MAVS, TRAF3 and TRAF6, which leads to ubiquitination-dependent degradation of every component of the MAVS/TRAF3/TRAF6 signalosome and subsequent potent inhibition of IFNs production. Consistently, OTUD1-deficient mice produce more antiviral cytokines and are more resistant to RNA virus infection. Our findings reveal a novel immune evasion mechanism exploited by RNA viruses, and elucidate a negative feedback loop of MAVS/TRAF3/TRAF6 signaling mediated by the OTUD1-Smurf1 axis during RNA virus infection.

## Author summary

Extensive studies have demonstrated redundant roles of multiple antiviral signaling pathways in host defense against viral infection. However, the strategies viruses have evolved to efficiently antagonize the complementary effect of alternative IFNs-inducing signaling remain largely unknown. Here we report that RNA viruses specifically promote the expression of the deubiquitinase OTUD1. RNA viruses-induced OTUD1 upregulates intracellular Smurf1 protein levels by deubiquitination modification, and promotes the binding of Smurf1 to MAVS, TRAF3 and TRAF6, which leads to degradation of every component of the MAVS/TRAF3/TRAF6 signalosome and inhibition of IFNs production. Our finding identifies a potent negative regulation of innate antiviral response, which could be crucial for RNA viruses to establish efficient infection at the early stage.

## Introduction

The innate immune response is the first line of host defense against viral infection. Viral nucleic acids can be recognized by host pattern recognition receptors (PRRs), including RIG-I-like receptors (RLRs), Toll-like receptors (TLRs) and cytosolic dsDNA sensors (STING) [1–5]. PRRs trigger antiviral signaling and result in the production of type I interferons (IFNs) and proinflammatory cytokines, which are central to the efficient host defense against viral infection [3,4,6]. RLRs (including RIG-I and MDA5) are mainly responsible for the recognition of cytosolic RNAs from RNA viruses [5,7]. RLRs recognize viral RNAs through the RNA helicase domain (RLD), and then interact with the mitochondrial antiviral signaling protein MAVS [7,8]. MAVS further recruits the tumor necrosis factor receptor-associated factor (TRAF) family proteins to form a key signaling platform, which triggers the IFNs antiviral response through activation of two important signaling pathways, type I IFNs (IFNα/β)-mediated antiviral IFN-stimulated genes (ISGs) signaling and NF-κB-mediated proinflammatory signaling [9–14].

MAVS is also known as IPS-1, Cardif or VISA [7,8,15,16]. Upon binding with viral RNAs, RIG-I activates MAVS by promoting MAVS polymerization on the mitochondrial surface [17,18]. MAVS polymers, as the central platform, recruit both TRAF3 and TRAF6 to form the MAVS/TRAF3/TRAF6 signalosome, which is essential to activate downstream antiviral signaling [18–21]. The MAVS/TRAF3/TRAF6 signalosome either interacts with a complex containing TBK1 and IKK to activate the transcription factor IRF3 and subsequent type I IFNs production, or interacts with IKKα/β/γ complex to activate NF-κB and produce downstream proinflammatory cytokines [18]. TRAF3 and TRAF6 belong to the TRAF family of ring-finger ubiquitin E3 ligases. TRAF3 regulates viral infection-triggered induction of IFNβ [22,23]. However, it was reported that in TRAF3-deficient MEFs RNA viruses can normally activate IRF3 and induce a modestly reduced level of IFNβ [18,24], suggesting that the function of TRAF3 can be substituted by other antiviral signaling for IFNs induction during viral infection. Interestingly, TRAF6 was also able to activate IRF3 and induce IFNs during viral infection [18]. Moreover, TRAF6 can act redundantly with TRAF2/5 to promote IRF3 and IKK activation by more complex mechanisms [18]. These studies indicate that TRAF family members (e.g. TRAF3 and TRAF6) could play redundant roles in inducing IFNs antiviral responses. However, whether and how viruses have evolved strategies to efficiently antagonize the complementary effect of various IFNs antiviral signaling remain largely unexplored.

Recently, some studies reported that negative regulation of MAVS levels by ubiquitin-proteasome system results in the inhibition of antiviral signaling. For example, PCBP1 and PCBP2 recruit Itch to induce K48-linked ubiquitination and degradation of MAVS [25,26]. In addition, viruses have evolved some strategies to antagonize MAVS-mediated antiviral signaling. Hepatitis B virus can induce ubiquitination on lysine 136 and degradation of MAVS through its X protein [27]. However, the deubiquitinases that regulate ubiquitination and levels of MAVS remain unexplored. Thus far, several ubiquitin E3 ligases and deubiquitinases have been reported to be associated with the activity or stability of TRAF3 and TRAF6. Cellular apoptosis inhibitors cIAP1 and cIAP2 induce K63-linked ubiquitination of TRAF3 and promote virus-triggered activation of NF-κB and IRF3 [28]. The deubiquitinases OTUB1/2 [29,30], UCHL1 [31], MYSM1 [32] and DUBA [33] inhibit K63-linked ubiquitination of TRAF3 or TRAF6 and negatively regulate IFNs production during viral infection. USP25 has been shown to enhance the stability of TRAF3 and TRAF6 [34]. However, how viral infection induces some common mechanisms to negatively regulate the stability of MAVS/TRAF3/TRAF6 signalosome remains to be illuminated.

In the present study, we report that the OTU deubiquitinase 1 (OTUD1) negatively regulates RNA virus-triggered production of type I IFNs and proinflammatory cytokines. OTUD1-deficient mice are more resistant to RNA virus infection by producing more type I IFNs. We further uncover that RNA viruses, but not DNA viruses, promote *Otud1* expression through the NF-κB signaling pathway. And OTUD1 upregulates Smurf1 protein levels by removing ubiquitination of Smurf1. Interestingly, RNA virus infection promotes the binding of Smurf1 to MAVS, TRAF3 and TRAF6, which results in ubiquitination and proteasome-dependent degradation of every component of the MAVS/TRAF3/TRAF6 signalosome. Our findings reveal a potent negative regulation of innate antiviral immune response by the OTUD1-Smurf1 axis-mediated downregulation of the MAVS/TRAF3/TRAF6 signalosome.

## Results

### OTUD1 is involved in the regulation of RNA virus-induced production of type I IFNs and proinflammatory factors

**Fig 1:**
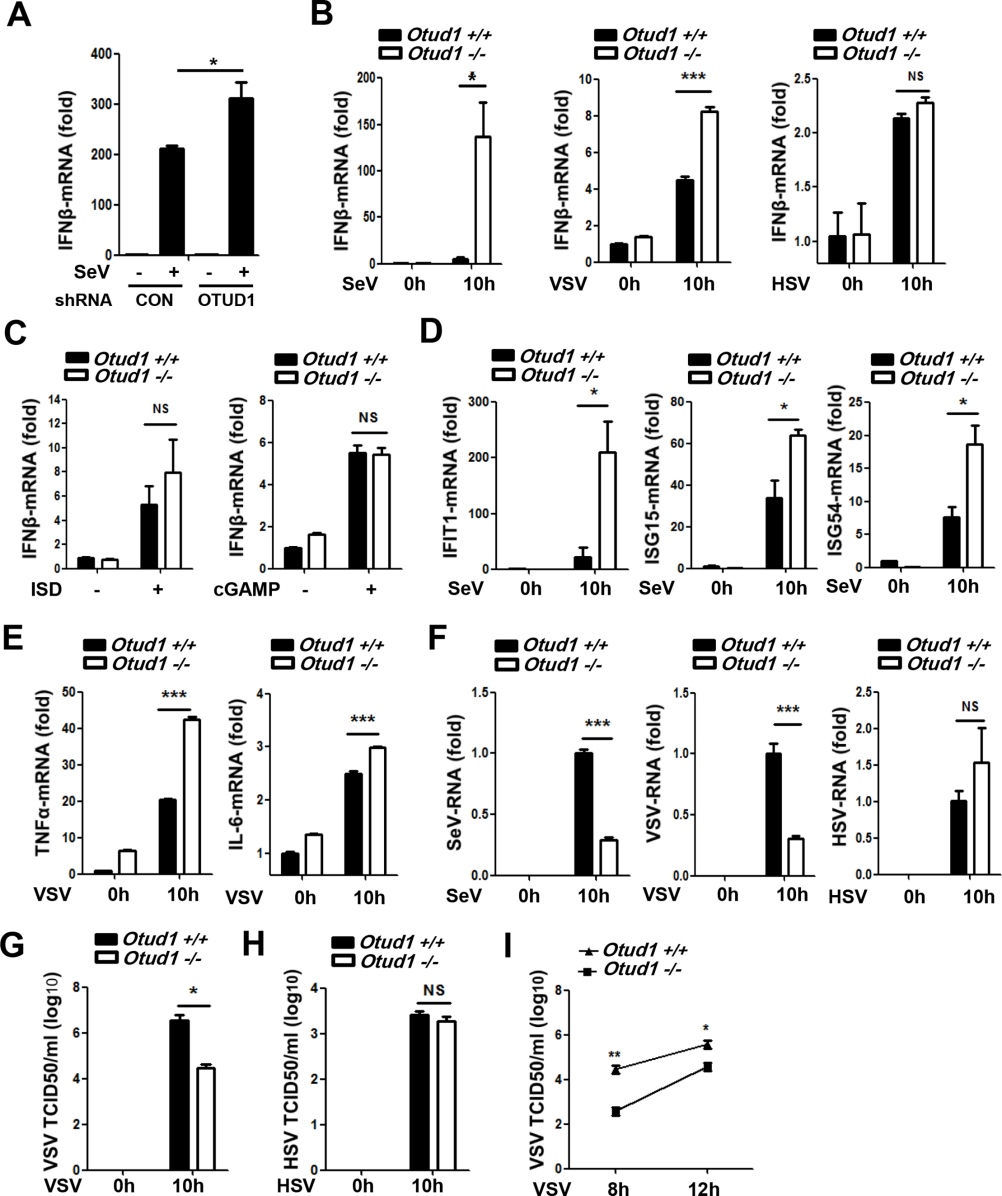
OTUD1 is involved in the regulation of RNA virus-induced production of type I IFNs and proinflammatory factors. (A) Relative mRNA expression of IFNβ in HEK293T cells transfected with control shRNAs (shCON) or OTUD1 shRNAs (shOTUD1) and then infected by SeV for 10 hr. The data were shown as fold change normalized to that of uninfected cells in the shCON group. (B) *Otud1*^+/+^ or *Otud1*^-/-^ MEFs were infected with SeV, or VSV, or HSV. 10 hr after infection, IFNβ mRNAs were analyzed by quantitative real time PCR (q-PCR). The data were shown as fold change normalized to that in uninfected *Otud1*^+/+^ MEFs. (C) Q-PCR analysis of IFNβ mRNA expression in *Otud1*^+/+^ or *Otud1*^-/-^ MEFs stimulated by ISD (2 μg/ml) or cGAMP (1 μg/ml) for 10 hr. The data were shown as fold change normalized to that in unstimulated *Otud1*^+/+^ MEFs. (D) IFIT1, ISG15 and ISG54 mRNA levels in *Otud1*^+/+^ or *Otud1*^-/-^ MEFs infected with SeV for 10 hr were analyzed by q-PCR. The data were shown as (B). (E) *Otud1*^+/+^ or *Otud1*^-/-^ MEFs were infected with VSV for 10 hr. The relative mRNA amounts of TNFα and IL-6 were determined by q-PCR. The data were shown as (B). (F) *Otud1*^+/+^ or *Otud1*^-/-^ MEFs were infected with SeV, or VSV, or HSV for 10 hr and then viral RNAs were analyzed by q-PCR. The data were shown as (B). (G and H) *Otud1*^*+/+*^ or *Otud1*^*-/-*^ MEFs were infected with VSV (G) or HSV (H) for 10 hr. Viral titers in supernatants were tested by TCID50 assay. (I) Determination of VSV titers in supernatants of *Otud1*^*+/+*^ or *Otud1*^*-/-*^ MEFs infected with VSV for the indicated time by TCID50 assay. NS: not significant (*P*>0.05). *P<0.05, **P<0.01 and ***P<0.001. Error bars represent the mean and s.d. of three independent experiments.

In a screen of deubiquitinases expression cloning library [35,36], we found that the deubiquitinase OTUD1 could inhibit RNA virus-induced production of type I IFNs. To further study the effect of OTUD1 on type I IFNs production, endogenous OTUD1 was knocked down and IFNβ mRNA levels after Sendai virus (SeV) infection were analyzed. The result showed that knockdown of OTUD1 promoted SeV-induced IFNβ production (Fig 1A and S1A Fig). In accordance with this response, the activity of promoters containing IFNs-stimulated response element (ISRE) was enhanced by OTUD1 knockdown (S1B Fig). And exogenous expression of OTUD1 lowered SeV-induced ISRE promoter activity (S1C Fig). To confirm the role of OTUD1 in the regulation of the production of type I IFNs, we infected mouse embryonic fibroblasts (MEFs) from *Otud1*^*+/+*^ and *Otud1*^*-/-*^ mice with either RNA viruses including SeV, vesicular stomatitis virus (VSV) and influenza A virus H1N1 (PR/8/34), or DNA viruses herpes simplex virus (HSV). The levels of IFNβ mRNA in *Otud1*^*-/-*^ MEFs were much higher than that in *Otud1*^*+/+*^ MEFs during infection of SeV, or VSV (Fig 1B), or H1N1 (S1D Fig). Interestingly, OTUD1 deletion did not affect production of IFNβ mRNA induced by HSV infection (Fig 1B). Given that HSV, as a DNA virus, majorly activates the STING (stimulator of interferon genes) signaling pathway, we employed another two specific stimulators, ISD (interferon stimulatory DNA) and cGAMP, for the DNA-STING signaling. The result showed that OTUD1 deficiency did not significantly affect IFNβ production stimulated by both ISD and cGAMP (Fig 1C). Together, these findings indicate that OTUD1 is involved in IFNs induction by RNA viruses, but not DNA-STING signaling. Interestingly, when *Otud1*^*+/+*^ and *Otud1*^*-/-*^ MEFs were stimulated by either Poly(I:C) (a TLR3 activator) or LPS (lipopolysaccharide, a TLR4 activator), we found that IFNβ induction by either Poly(I:C) or LPS was remarkably upregulated in *Otud1*^*-/-*^ cells (S1E Fig), indicating that OTUD1 could play roles in IFNs induction by at least some of TLRs signaling.

Consistent with the data from RNA viruses, *Otud1*^-/-^ mice had more IFNβ protein in the sera than did *Otud1*^*+/+*^ mice in response to VSV infection (S1F Fig). Additionally, *Otud1*^*-/-*^ MEFs displayed more expression of interferon-stimulated genes (ISGs) including IFIT1, ISG15 and ISG54 during SeV infection, as compared with their wild-type counterparts (Fig 1D). Similarly, knockdown of OTUD1 by shRNAs promoted SeV-induced mRNA expression of ISGs (S1G Fig). Interestingly, OTUD1 deficiency also promoted RNA virus-induced production of proinflammatory factors such as TNFα and IL-6 in either MEFs (Fig 1E) or mouse primary liver cells (S1H and S1I Fig), suggesting that OTUD1 could target certain components upstream of RLRs-mediated antiviral signaling. Finally, we analyzed the effect of OTUD1 deficiency on viral infection. The result showed that OTUD1 deletion markedly downregulated viral RNA levels in MEFs cells infected with both SeV and VSV (Fig 1F). However, OTUD1 deficiency did not affect HSV RNA levels (Fig 1F). Furthermore, viral titers were determined by the 50% tissue culture infectious dose (TCID50) assay. We found that OTUD1 deletion lowered RNA virus VSV viral titers (Fig 1G and 1I), but did not downregulate HSV viral titers (Fig 1H). Taken together, these results suggest that OTUD1 is involved in host defense against RNA viruses.

### OTUD1 deficiency protects mice from RNA virus infection

**Fig 2:**
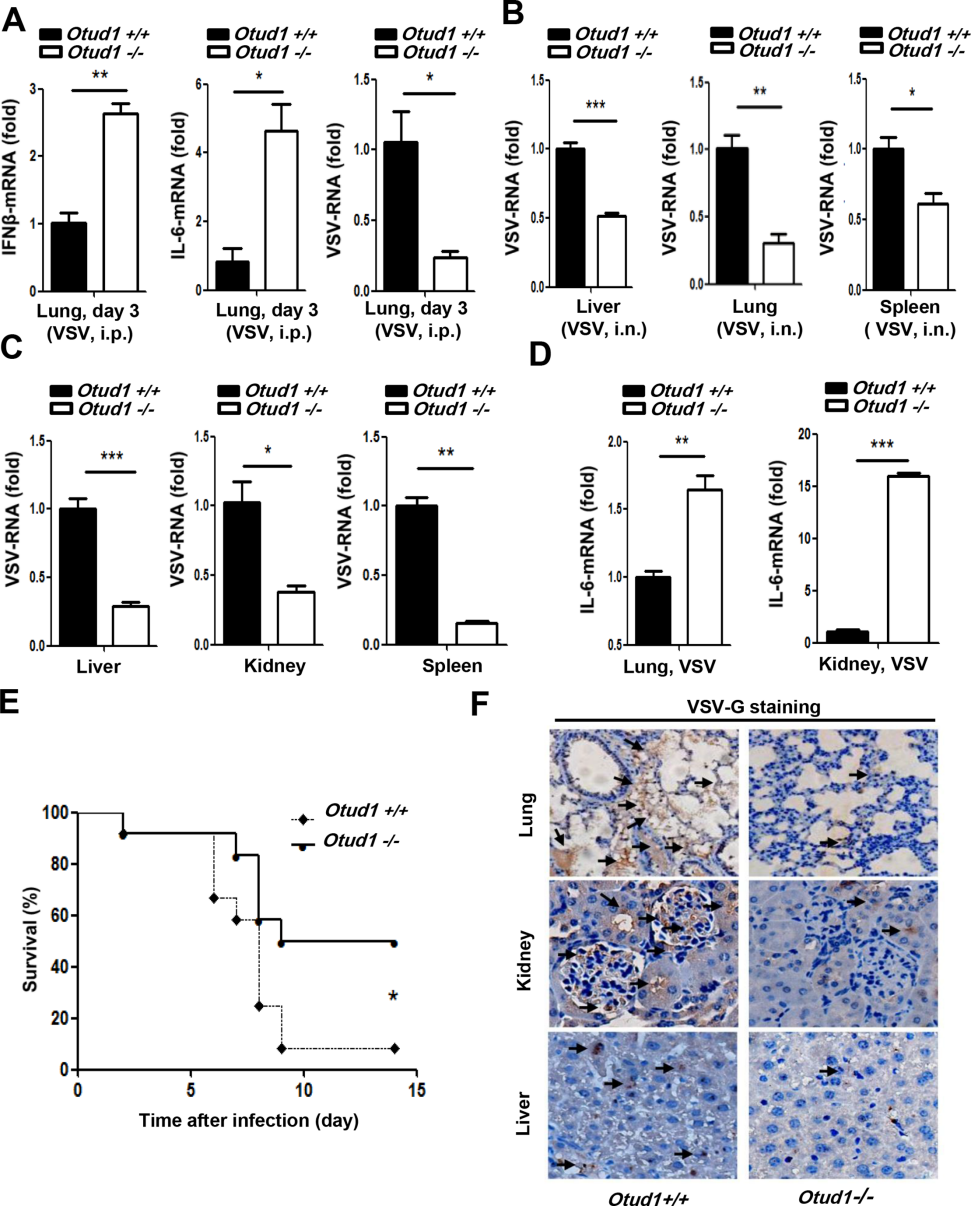
OTUD1 deficiency protects mice from RNA virus infection. (A) Q-PCR analysis of IFNβ (left), IL-6 (middle) and VSV (right) mRNA expression in the lungs of *Otud1*^+/+^ or *Otud1*^-/-^ mice (n = 5 per group) 3 days after intraperitoneal injection with VSV (2X10^8^ pfu per mouse). The data were shown as fold change normalized to that in *Otud1*^+/+^ mice. (B) Q-PCR analysis of VSV RNA expression in the liver (left), lung (middle) and spleen (right) of *Otud1*^+/+^ or *Otud1*^-/-^ mice (n = 5 per group) with intranasal infection by VSV (2X10^7^ pfu per mouse) for 3 days. The data were shown as (A). (C) Q-PCR analysis of VSV RNAs expression in the organs of *Otud1*^+/+^ or *Otud1*^-/-^ mice (n = 4 per group) 14 days after intraperitoneal injection with VSV (2X10^8^ pfu per mouse). The data were shown as (A). (D) Q-PCR analysis of IL-6 mRNA expression in the organs of *Otud1*^+/+^ or *Otud1*^-/-^ mice (n = 4 per group) 14 days after intraperitoneal injection with VSV (2X10^8^ pfu per mouse). The data were shown as (A). (E) Survival of *Otud1*^+/+^ or *Otud1*^-/-^ mice (n = 12 per group) given intranasal infection of VSV (2X10^7^ pfu per mouse). *P<0.05. (F) Immunohistochemistry analysis of VSV encoded VSVG protein in the organs from *Otud1*^+/+^ or *Otud1*^-/-^ mice with intranasal VSV (2X10^7^ pfu per mouse) infection for 3 days. *P<0.05, **P<0.01 and ***P<0.001 (unpaired *t*-test (A-D) or Gehan-Breslow-Wilcoxon test (E)). Error bars represent the mean and s.d. of three independent experiments.

To investigate the role and functional importance of OTUD1 in host antiviral response in vivo, we challenged wild-type and *Otud1*^*-/-*^ mice with VSV using intraperitoneal injection. At day 3 after VSV infection, *Otud1*^*-/-*^ mice produced much higher expression of IFNβ and IL-6 mRNAs (Fig 2A), and had much lower viral RNAs (Fig 2A) in their lung tissue than did *Otud1*^*+/+*^ mice. Consistent with the results obtained by intraperitoneal VSV injection, *Otud1*^*-/-*^ mice with VSV intranasal infection had lower VSV loads at day 3 than did *Otud1*^*+/+*^ mice (Fig 2B). Furthermore, when viral infection was extended to 14 days, VSV loads in different organs from *Otud1*^*-/-*^ mice were significantly reduced compared to their wild-type counterparts (Fig 2C). Then we observed the expression of proinflammatory factor IL-6 mRNA in different organs from wild-type and *Otud1*^*-/-*^ mice. We found that the levels of IL-6 mRNA in organs from *Otud1*^*-/-*^ mice were much higher than that from *Otud1*^*+/+*^ mice (Fig 2D). We next challenged *Otud1*^*+/+*^ and *Otud1*^*-/-*^ mice with VSV and monitored their survival. The results showed that *Otud1*^*-/-*^ mice were more resistant to VSV infection in overall survival assays (Fig 2E). To directly observe the VSV infection in different organs from wild-type and *Otud1*^*-/-*^ mice, immunohistochemical staining for VSV encoded protein VSVG was carried out. The results showed less VSV staining in lung, kidney and liver tissue from *Otud1*^*-/-*^ mice, as compared with wild-type mice (Fig 2F). Collectively, our data suggest that *Otud1*^*-/-*^ mice possess more potent host defense against RNA viruses by promoting the induction of type I IFNs and proinflammatory cytokines.

### OTUD1 negatively regulates protein levels of MAVS, TRAF3, and TRAF6

**Fig 3:**
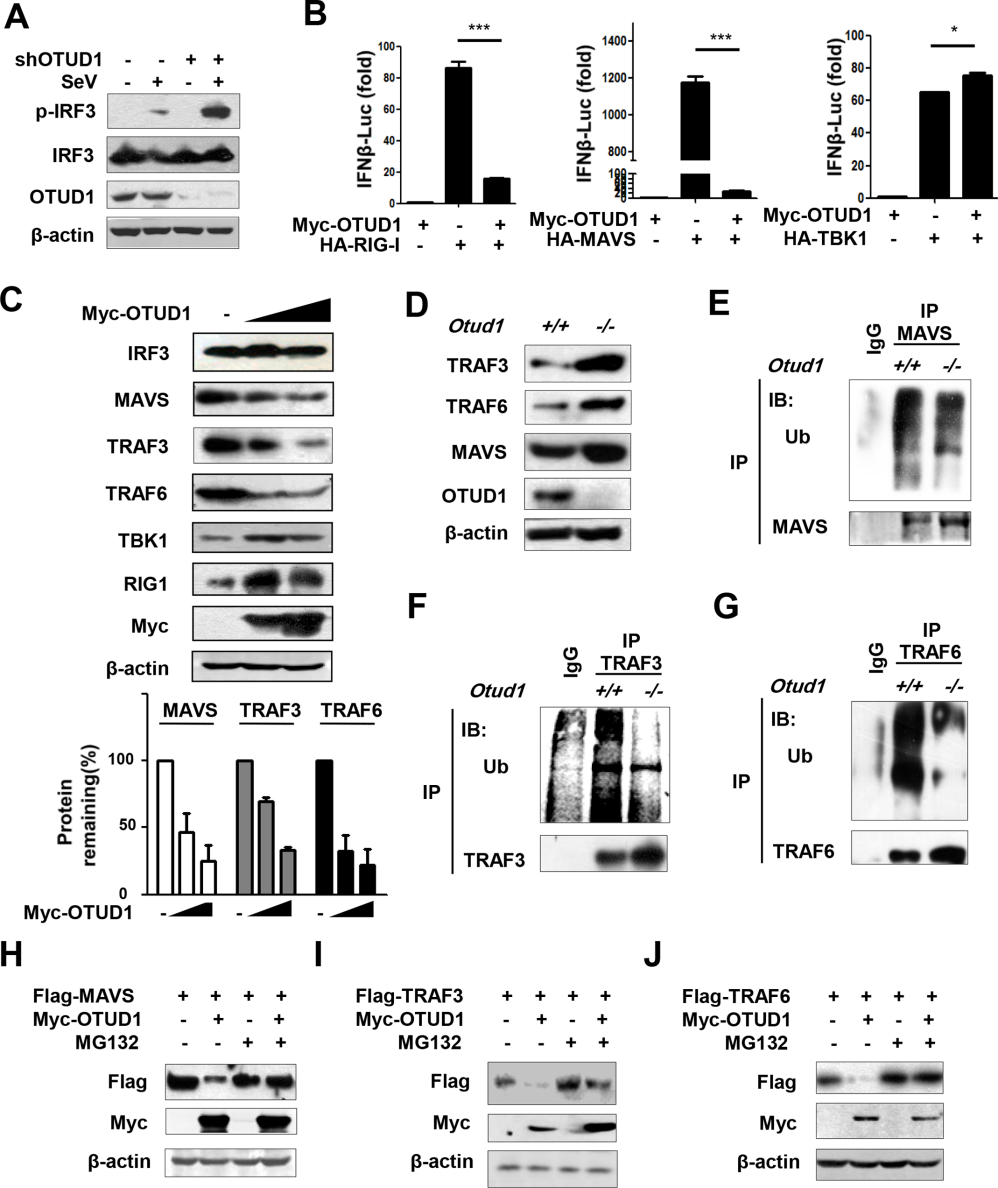
OTUD1 negatively regulates protein levels of MAVS, TRAF3, and TRAF6. (A) Immunoblot analysis of phosphorylated IRF3 (p-IRF3) in HeLa cells transfected with shRNAs plasmids (shCON or shOTUD1), and then infected with SeV for 12 hr. (B) HEK293T cells were transfected with vector or Flag-OTUD1, together with HA-RIG1, or HA-MAVS, or HA-TBK1. The IFNβ luciferase activity was analyzed after SeV infection. The data were shown as fold change normalized to that in cells without HA-RIG1/MAVS/TBK1. (C) HEK293T cells were transfected with increasing amounts of Myc-OTUD1. The levels of indicated proteins were analyzed by immunoblotting. The densitometry of the proteins (MAVS, TRAF3 and TRAF6) is presented relative to those of β–actin, and quantification of relative protein levels was from three independent experiments. (D) Immunoblot analysis of the indicated proteins in the *Otud1*^+/+^ or *Otud1*^-/-^ primary MEFs. (E-G) MAVS, TRAF3 and TRAF6 were immunoprecipitated from whole cell lysates of *Otud1*^+/+^ or *Otud1*^-/-^ MEFs. Ubiquitination levels of MAVS (E), TRAF3 (F) and TRAF6 (G) were detected by immunoblotting by a specific Ub antibody. (H-J) HEK293T cells were transfected with Myc-OTUD1, together with Flag-MAVS (H), or Flag-TRAF3 (I), or Flag-TRAF6 (J). Cells were either untreated or pretreated with MG132 (20 μM) for 4 hr before the harvest. The levels of Flag-MAVS (H), Flag-TRAF3 (I) and Flag-TRAF6 (J) were detected by immunoblotting as indicated. *P<0.05, **P<0.01 and ***P<0.001 (unpaired *t*-test). Data are representative of three independent experiments.

To uncover the mechanisms by which OTUD1 regulates antiviral innate immune response, we first analyzed the effect of OTUD1 on activation of IFNβ promoter during RNA virus infection. Results showed that knockdown of OTUD1 significantly enhanced activation of IFNβ promoter during SeV infection (S2A Fig), and overexpression of OTUD1 inhibited SeV-induced activation of IFNβ promoter (S2B Fig). Consistently, OTUD1 knockdown increased phosphorylated IRF3 levels induced by SeV infection (Fig 3A). Conversely, OTUD1 overexpression decreased the levels of phosphorylated IRF3 (S2C Fig) and IRF3 homodimers (S2D Fig) during viral infection. To further determine at what level in RLRs-mediated antiviral signaling pathway OTUD1 blocked activation of IFNβ promoter, three expression constructs (RIG-I, MAVS, or TBK1) were overexpressed in cells in the presence or absence of OTUD1. The results showed that OTUD1 overexpression dramatically inhibited IFNβ promoter activation driven by both RIG-I and MAVS (Fig 3B), whereas IFNβ promoter activation by TBK1 (Fig 3B) was not inhibited by OTUD1 coexpression. These data indicate that OTUD1 inhibits the IFNs antiviral response downstream of MAVS and upstream of TBK1.

To further identify the signaling proteins targeted by OTUD1, the interaction between OTUD1 and MAVS was first analyzed. Our data showed that OTUD1 was able to interact with MAVS (S3A Fig). MAVS is a key platform protein that majorly recruits TRAF3 and TRAF6 to form a signalosome. Therefore, we studied whether OTUD1 could interact with TRAF3 or TRAF6. Results showed that OTUD1 was also capable of interacting with both TRAF3 (S3B Fig) and TRAF6 (S3C Fig). Interestingly, we repeatedly observed that protein levels of MAVS (S3A and S4A Figs), TRAF3 (S3B and S4B Figs), and TRAF6 (S3C and S4C Figs) were noticeably decreased when OTUD1 was coexpressed. These data raise the question whether OTUD1 could regulate MAVS/TRAF3/TRAF6 signaling. To address this hypothesis, OTUD1 was gradually overexpressed in cells and the levels of endogenous signaling proteins in the antiviral signaling pathway were firstly determined. Our data showed that OTUD1 overexpression had no obvious effects on IRF3 and TBK1 protein levels, and increased RIG-I protein levels to some extent (Fig 3C). However, we noticed that OTUD1 overexpression gradually downregulated the levels of MAVS, TRAF3, and TRAF6 (Fig 3C), which is consistent with the observation that OTUD1 overexpression inhibits the induction of type I IFNs and proinflammatory cytokines. To confirm the effects of OTUD1 on MAVS/TRAF3/TRAF6 protein levels, we determined the levels of endogenous MAVS/TRAF3/TRAF6 in *Otud1*^*+/+*^ and *Otud1*^*-/-*^ MEFs. The results showed that cellular levels of MAVS, TRAF3, and TRAF6 in *Otud1*^*-/-*^ MEFs were higher than that in wild-type counterparts to some extent (Fig 3D). Together, these data suggest that OTUD1 negatively regulates protein levels of MAVS, TRAF3, and TRAF6.

Next, we hypothesize that OTUD1 affects protein stability of MAVS, TRAF3, and TRAF6. Endogenous MAVS, or TRAF3, or TRAF6 proteins from *Otud1*^*+/+*^ and *Otud1*^*-/-*^ MEFs were immunoprecipitated, and the ubiquitination levels were firstly determined by a specific ubiquitin antibody. We found that the ubiquitination levels of MAVS (Fig 3E), TRAF3 (Fig 3F), and TRAF6 (Fig 3G) were reduced in *Otud1*^-/-^ MEFs when comparing to *Otud1*^*+/+*^ MEFs. And overexpression of OTUD1 promoted K48-linked ubiquitination of MAVS/TRAF3/TRAF6 (S4E Fig), suggesting that OTUD1 promotes ubiquitination and degradation of MAVS/TRAF3/TRAF6. Furthermore, the proteasome inhibitor MG132 was used to block ubiquitin-proteasome degradation. We noticed that endogenous MAVS protein is relatively stable, which is consistent with endogenous OTUD1 protein stability, as shown by our observation that the pretreatment of cells with MG132 for 8 hr and 12 hr did not significantly upregulate protein levels of endogenous OTUD1 and MAVS (S4F Fig). However, when OTUD1 was overexpressed, the levels of MAVS were markedly downregulated (Fig 3H). Similarly, OTUD1 overexpression lowered protein levels of TRAF3 (Fig 3I) and TRAF6 (Fig 3J). In the presence of MG132, the ability of OTUD1 to downregulate MAVS (Fig 3H), or TRAF3 (Fig 3I), or TRAF6 (Fig 3J) was largely inhibited. Collectively, these results suggest that OTUD1 plays a role in promoting proteasome-dependent degradation of MAVS/TRAF3/TRAF6.

### Both Smurf1 and the deubiquitinase activity of OTUD1 are required for OTUD1-mediated downregulation of MAVS/TRAF3/TRAF6 and inhibition of interferon antiviral response

**Fig 4:**
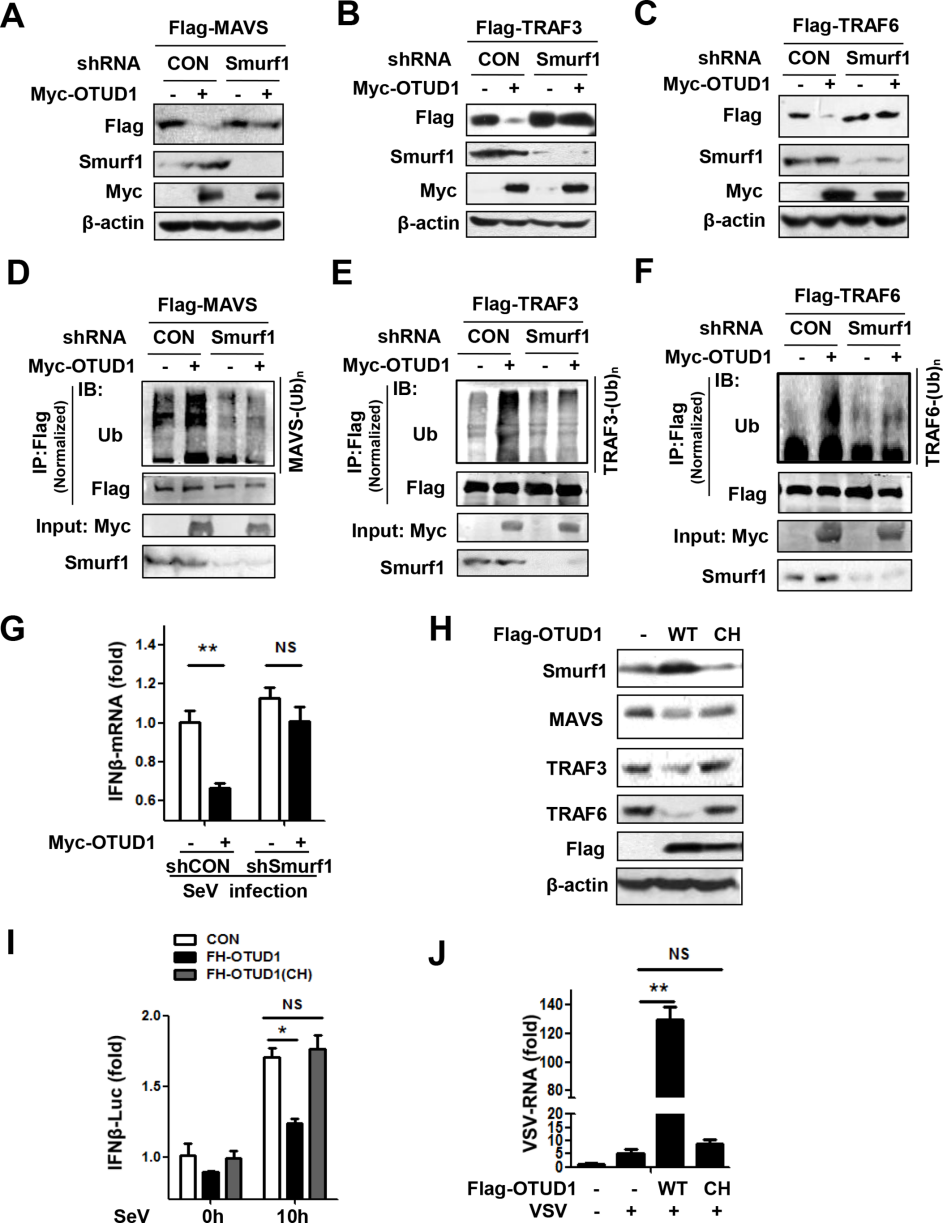
Both Smurf1 and the deubiquitinase activity of OTUD1 are required for OTUD1-mediated downregulation of MAVS/TRAF3/TRAF6 and inhibition of interferon antiviral response. (A-C) HEK293T cells were transfected with Flag-MAVS (A), or Flag-TRAF3 (B), or Flag-TRAF6 (C), together with or without shSmurf1 and Myc-OTUD1 as indicated. The whole cell lysates were analyzed by immunoblotting. (D-F) HEK293T cells were transfected with Flag-MAVS (D), or Flag-TRAF3 (E), or Flag-TRAF6 (F), together with or without shSmurf1 and Myc-OTUD1 as indicated. Immunoprecipitation and immunoblot analysis were carried out as indicated. (G) HEK293T cells were transfected with or without shSmurf1 and Myc-OTUD1 as indicated. After 48 hr, cells were infected with SeV for 12 hr. Relative mRNA expression of IFNβ was determined by q-PCR. The data were shown as fold change normalized to that in the shCON group without Myc-OTUD1. (H) Immunoblot analysis of Smurf1, MAVS, TRAF3 and TRAF6 proteins in whole cell lysates of HEK293T cells transfected with empty vector, wild-type Flag-OTUD1, or catalytically inactive Flag-OTUD1 (CH). (I) *Otud1*^-/-^ MEFs were transfected with empty vector, Flag-OTUD1, or Flag-OTUD1 (CH), and then infected with SeV for 12 hr. Relative IFNβ mRNA expression was determined by q-PCR. The data were shown as fold change normalized to that in the uninfected control group. (J) HEK293T cells were transfected with empty vector, or Flag-OTUD1 wild type, or Flag-OTUD1 (CH), and then infected with VSV for 12 hr. Relative VSV viral RNA expression was analyzed by q-PCR. The data were shown as (I). *P<0.05 and **P<0.01, NS, not significant (*P*>0.05) (unpaired *t*-test). Error bars represent the mean and s.d., and all data are representative of three independent experiments.

OTUD1 is the member of the deubiquitinases, which are supposed to remove ubiquitination from protein substrates and upregulate protein levels. Our above data demonstrated that OTUD1 promoted ubiquitination of MAVS/TRAF3/TRAF6, and downregulated their protein levels, suggesting that MAVS, TRAF3, and TRAF6 are not the direct targets of OTUD1 as a deubiquitinase. Therefore, we asked whether OTUD1 could upregulate certain ubiquitin E3 ligases, which directly regulate ubiquitination and degradation of MAVS/TRAF3/TRAF6. So far, several ubiquitin E3 ligases have been reported to be involved in promoting TRAF3/6 ubiquitination. Both cIAP1 and cIAP2 promoted only K63-linked ubiquitination of TRAF3 [28], which should not be the ubiquitin E3 ligase resulting in TRAF3 downregulation. It was reported that Ndfip1 interacted with the E3 ligase Smurf1 and promoted MAVS degradation [37]. In addition, the E3 ligase Smurf2 also induced ubiquitination and degradation of MAVS [38]. We further found that OTUD1 overexpression upregulated protein level of Smurf1, rather than Smurf2 (S4D Fig). Therefore, we tried to analyze whether Smurf1 could be required for OTUD1-mediated downregulation of MAVS. Our data showed that knockdown of Smurf1 significantly inhibited OTUD1-mediated MAVS downregulation (Fig 4A). Interestingly, Smurf1 knockdown also remarkably blocked OTUD1-mediated downregulation of both TRAF3 (Fig 4B) and TRAF6 (Fig 4C). Consistently, knockdown of Smurf1 inhibited OTUD1-induced ubiquitination of MAVS (Fig 4D), TRAF3 (Fig 4E) and TRAF6 (Fig 4F). The role of Smurf1 in regulating TRAF3/TRAF6 protein levels during viral infection remains unknown so far, although Smurf1 was reported to be able to bind to and ubiquitinate exogenous Myc-TRAFs family members in HEK293T cells [39]. Here, our results indicate that OTUD1 could utilize Smurf1 to downregulate MAVS/TRAF3/TRAF6 proteins and restrict IFNs production during viral infection. Therefore, we further observed the role of Smurf1 in OTUD1-mediated inhibitory effect on IFNs production during viral infection. Our data showed that overexpression of OTUD1 inhibited the production of IFNβ in response to SeV infection, whereas knockdown of Smurf1 largely blocked the negative regulation of OTUD1 on IFNβ production (Fig 4G), indicating that Smurf1 is required for OTUD1-mediated inhibition of interferon antiviral response.

We next investigate whether the deubiquitinase activity of OTUD1 is required for downregulation of MAVS/TRAF3/TRAF6 and interferon antiviral response. According to the conservative sites of the deubiquitinase activity of OTU family [40], we constructed OTUD1-C320A-H431Q (CH) mutant. As compared with OTUD1-wild type constructs, OTUD1-CH mutants lost the ability to downregulate MAVS, TRAF3, and TRAF6 (Fig 4H). In addition, we observed that OTUD1-CH mutants were unable to upregulate Smurf1 protein as well (Fig 4H). These results indicate that the deubiquitinase activity of OTUD1 is required for Smurf1 upregulation and MAVS/TRAF3/TRAF6 downregulation.

Given that OTUD1 downregulated MAVS/TRAF3/TRAF6 protein levels, we next questioned whether OTUD1 could inhibit MAVS/TRAF3/TRAF6 signalosome-mediated IFNs production and antiviral activity. During infection with SeV, overexpression of OTUD1-wild type obviously lowered the activity of IFNβ promoter, whereas overexpression of OTUD1-CH mutant completely had no effect on the IFNβ promoter activity (Fig 4I), suggesting that the deubiquitinase activity of OTUD1 is important for OTUD1-mediated inhibition of IFNβ production during viral infection. Moreover, we investigated the effect of the deubiquitinase activity of OTUD1 on viral infection. The results showed that overexpression of OTUD1-wild type dramatically promoted VSV infection (Fig 4J). In contrast, overexpression of OTUD1-CH mutant had no effect on VSV infection (Fig 4J). Taken together, these results suggest that both Smurf1 and the deubiquitinase activity of OTUD1 are required for the downregulation of MAVS/TRAF3/TRAF6 signalosome proteins, which results in the OTUD1-mediated inhibition of interferon antiviral response.

### OTUD1 regulates ubiquitination and protein levels of Smurf1

**Fig 5:**
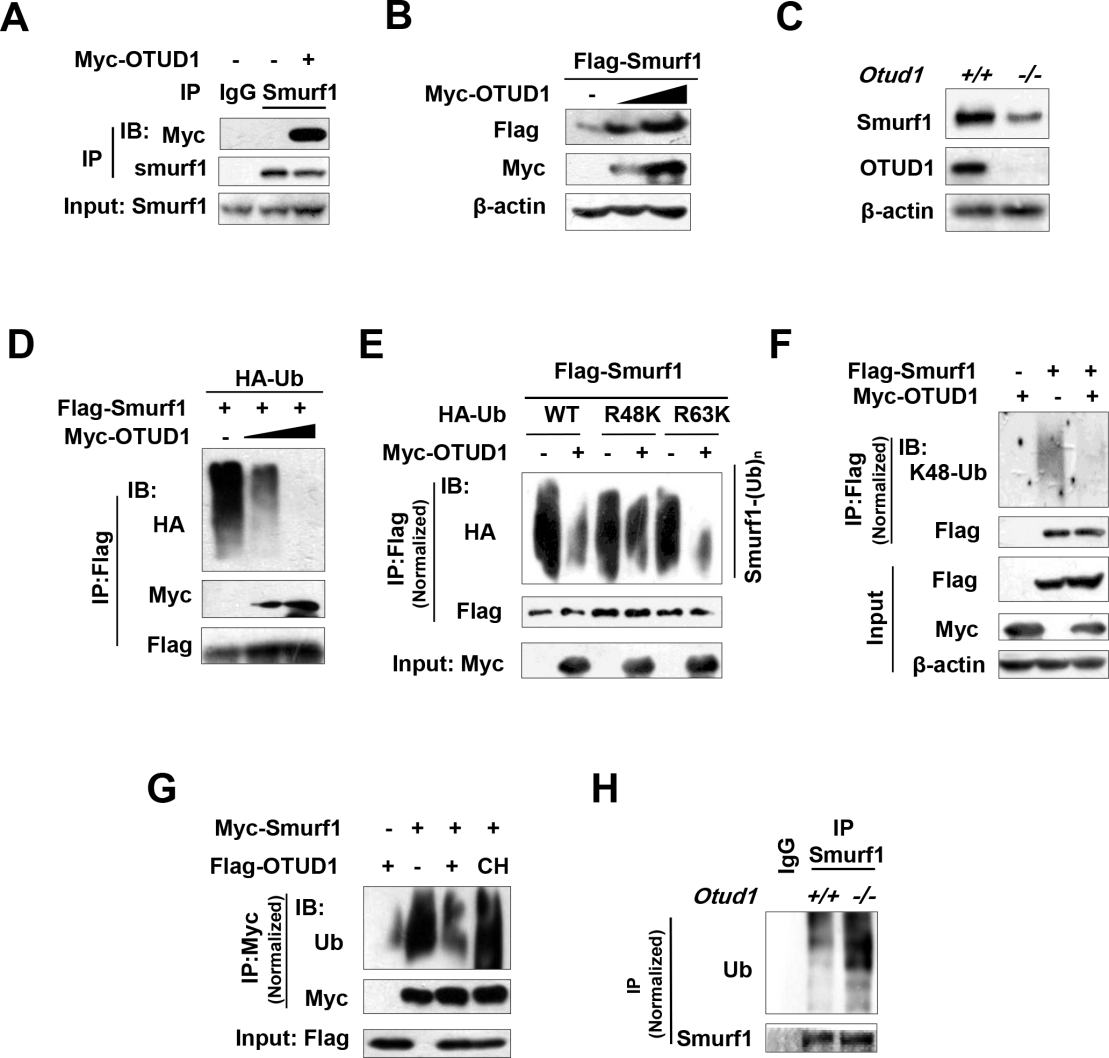
OTUD1 regulates ubiquitination and protein levels of Smurf1. (A) Smurf1 immunoprecipitation was performed from HEK293T cells expressing with empty vector or Myc-OTUD1. Myc-OTUD1 was detected by immunoblotting as indicated. (B) HEK293T cells were transfected with Flag-Smurf1 and various doses of Myc-OTUD1. The levels of Flag-Smurf1 were examined as shown. (C) Immunoblot analysis of Smurf1 protein in the *Otud1*^+/+^ or *Otud1*^-/-^ MEFs. (D) HEK293T cells were transfected with Flag-Smurf1, HA-Ub, and various doses of Myc-OTUD1. Immunoprecipitation and immunoblotting were performed as indicated. (E) HEK293T cells were transfected with Flag-Smurf1 and Myc-OTUD1, together with HA-Ub or HA-Ub-R48K (K48-only) or HA-R63K (K63-only). Immunoprecipitation and immunoblot analysis were carried out as indicated. (F) HEK293T cells were transfected with Flag-Smurf1 and Myc-OTUD1. Flag-Smurf1 was immunoprecipitated, and K48-linked ubiquitination levels of Flag-Smurf1 were analyzed by a specific K48-Ub antibody. (G) HEK293T cells were transfected with Myc-Smurf1, together with Flag-OTUD1 wild type or Flag-OTUD1(CH) mutant. Myc-Smurf1 was immunoprecipitated by a Myc antibody, and then ubiquitination levels of Myc-Smurf1 were analyzed by a specific Ub antibody. (H) Ubiquitination levels of Smurf1 in *Otud1*^+/+^ or *Otud1*^-/-^ MEFs were analyzed as indicated. Data are representative of three independent experiments.

The aforementioned results prove that Smurf1 is important for OTUD1-mediated inhibition of interferon antiviral response. We next sought to determine whether OTUD1 could regulate Smurf1. We found that Smurf1 was able to interact with OTUD1 (Fig 5A). Overexpression of OTUD1 upregulated exogenous Smurf1 protein levels in a dose-dependent manner (Fig 5B). When endogenous Smurf1 protein was analyzed in *Otud1*^*+/+*^ and *Otud1*^*-/-*^ MEFs, we found that the level of endogenous Smurf1 in *Otud1*^*-/-*^ MEFs was noticeably lower than that in *Otud1*^*+/+*^ MEFs (Fig 5C), suggesting that OTUD1 is a positive regulator of Smurf1 protein levels.

Furthermore, the effect of OTUD1 on Smurf1 ubiquitination was determined. The results showed that overexpression of OTUD1 significantly removed polyubiquitination of Smurf1 (Fig 5D). Next, we analyzed the deubiquitination types of Smurf1 mediated by OTUD1 using two ubiquitin mutants, Ub-R48K (all lysines in Ub are mutated to arginines except lysine 48 residue) and Ub-R63K (all lysines in Ub are mutated to arginines except lysine 63 residue). We found that OTUD1 overexpression decreased both K48-linked and K63-linked ubiquitination of Smurf1 (Fig 5E). By a specific K48-linked ubiquitin antibody, we confirmed that OTUD1 was capable of removing K48-linked ubiquitination of Smurf1 (Fig 5F). In addition, the deubiquitination effect of OTUD1 on Smurf1 protein was dependent on the deubiquitinase activity of OTUD1 (Fig 5G). Furthermore, we analyzed ubiquitination of endogenous Smurf1 in *Otud1*^*+/+*^ and *Otud1*^*-/-*^ MEFs. The results showed that OTUD1 deficiency upregulated ubiquitination levels of endogenous Smurf1 protein (Fig 5H). Thus, these data demonstrate that OTUD1 positively regulates cellular levels of Smurf1 by deubiquitination effects.

### RNA virus infection promotes NF-κB-dependent expression of OTUD1 that upregulates Smurf1 protein levels

**Fig 6:**
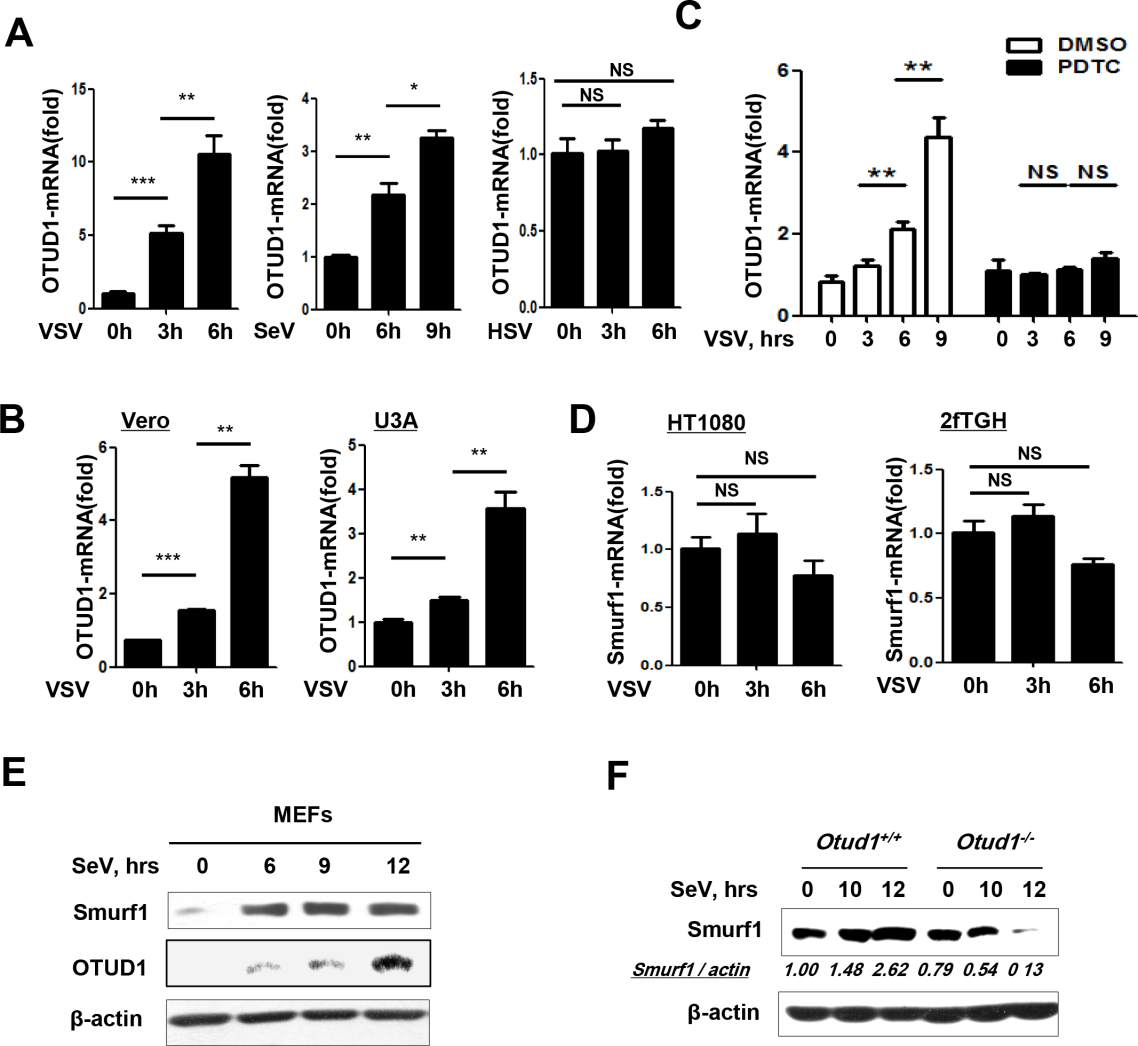
RNA virus infection promotes NF-κB-dependent expression of OTUD1, and which upregulates levels of Smurf1. (A) Q-PCR analysis of OTUD1 mRNA expression in HT1080 (left) infected with VSV (MOI = 3) or 2fTGH (middle and right) infected with SeV (MOI = 3) or HSV (MOI = 3). The data were shown as fold change normalized to that in uninfected cells. (B) Q-PCR analysis of OTUD1 mRNA expression in Vero (left) and U3A (right) cells infected with VSV (MOI = 3). The data were shown as (A). (C) Q-PCR analysis of OTUD1 mRNA expression in 2fTGH cells pretreated with PDTC (NF-κB inhibitor, 10 μM) for 1 hr before VSV (MOI = 3) infection. The data were shown as fold change normalized to that in uninfected control cells. (D) Q-PCR analysis of Smurf1 mRNA expression in HT1080 (left) and 2fTGH (right) cells infected with VSV (MOI = 3). The data were shown as (A). (E) Immunoblot analysis of Smurf1 and OTUD1 proteins in the whole cell lysates from MEFs cells infected with SeV (MOI = 3) for the indicated times. (F) *Otud1*^+/+^or *Otud1*^-/-^ MEFs were infected with SeV (MOI = 3) for the indicated times. The level of Smurf1 protein was determined by Immunoblotting. NS, not significant (*P*>0.05); *P<0.05, **P<0.01 and ***P<0.001 (unpaired *t*-test). Error bars represent the mean and s.d., and all data are representative of three independent experiments.

We next sought to determine how viral infection affects the levels of OTUD1, Smurf1 and MAVS/TRAF3/TRAF6, and whether the regulation signaling of OTUD1-Smurf1-MAVS/TRAF3/TRAF6 occurs during viral infection. We found that VSV infection upregulated expression of OTUD1 mRNA in human fibrosarcoma cells HT1080 (Fig 6A, left panel) and 2fTGH (S5A Fig). Similarly, SeV infection also promoted expression of OTUD1 mRNA in a time-dependent manner (Fig 6A, middle panel). However, DNA viruses HSV did not upregulate OTUD1 mRNA expression in 2fTGH (Fig 6A, right panel) or MEFs (S5B Fig) at the early stage of infection. Besides HSV, another two stimulators for the STING signaling, ISD and cGAMP, did not activate OTUD1 mRNA expression either (S5D and S5E Fig). Consistently, as demonstrated before, OTUD1 cannot regulate IFNs induction by these three stimulators for the STING signaling (Fig 1B and 1C). Interestingly, we found that Poly(I:C), a TLR3-signaling stimulator, also induced OTUD1 mRNA expression (S5C Fig). Consistently, OTUD1 is able to regulate Poly(I:C)-induced IFNs production (S1E Fig). Based on all above observations, we think that activation of OTUD1 expression is an important trigger for the OTUD1-Smurf1-MAVS/TRAF3/TRAF6-IFNs signaling.

During RNA virus infection, two major activated pathways including IFNs signaling and NF-κB signaling induce activation and expression of thousands of downstream genes. Furthermore, we studied which signaling is responsible for upregulation of OTUD1 mRNA during RNA virus infection. We found that VSV infection also upregulated expression of OTUD1 mRNA in Vero cells (type I interferon-deficiency) and U3A cells (STAT1-deficiency) (Fig 6B). Using a NF-κB inhibitor PDTC, we found that VSV-induced upregulation of OTUD1 mRNA was significantly inhibited by pretreatment of cells with the NF-κB inhibitor (Fig 6C), suggesting that NF-κB signaling contributes to RNA virus-induced upregulation of OTUD1 mRNA. As to Smurf1 mRNA, VSV infection did not significantly affect the level of Smurf1 mRNA in HT1080 and 2fTGH cells within 6 hours after infection (Fig 6D). Collectively, these data suggest that during RNA virus infection OTUD1 mRNA was upregulated via virus-induced NF-κB signaling, whereas the level of Smurf1 mRNA remains relatively stable at the early stage of RNA virus infection.

The above results demonstrated that RNA virus infection did not significantly change the level of Smurf1 mRNA at the early stage. However, we found that SeV infection in HeLa cells obviously upregulated Smurf1 protein levels in a time-dependent manner (S5F Fig). This similar phenomenon of Smurf1 upregulation during RNA virus infection was also observed in other cell lines including MEFs (Fig 6E). Interestingly, there was a positive correlation between Smurf1 and OTUD1 protein levels during RNA virus infection, as shown in MEFs cells (Fig 6E). Moreover, when analyzing the changes of Smurf1 protein levels in *Otud1*^*+/+*^ and *Otud1*^*-/-*^ MEFs during SeV infection, we found that Smurf1 protein levels were gradually upregulated in *Otud1*^*+/+*^ MEFs, whereas SeV infection did not upregulate Smurf1 protein levels in *Otud1*^*-/-*^ MEFs (Fig 6F). Conversely, in *Otud1*^*-/-*^ MEFs Smurf1 protein was quite unstable and was rapidly downregulated during SeV infection (Fig 6F). Taken together, these results suggest that RNA viruses specifically upregulate OTUD1 expression, which increases the stability and levels of Smurf1 protein.

### RNA virus infection promotes the interaction between OTUD1 and Smurf1, and binding of Smurf1 to MAVS/TRAF3/TRAF6

**Fig 7:**
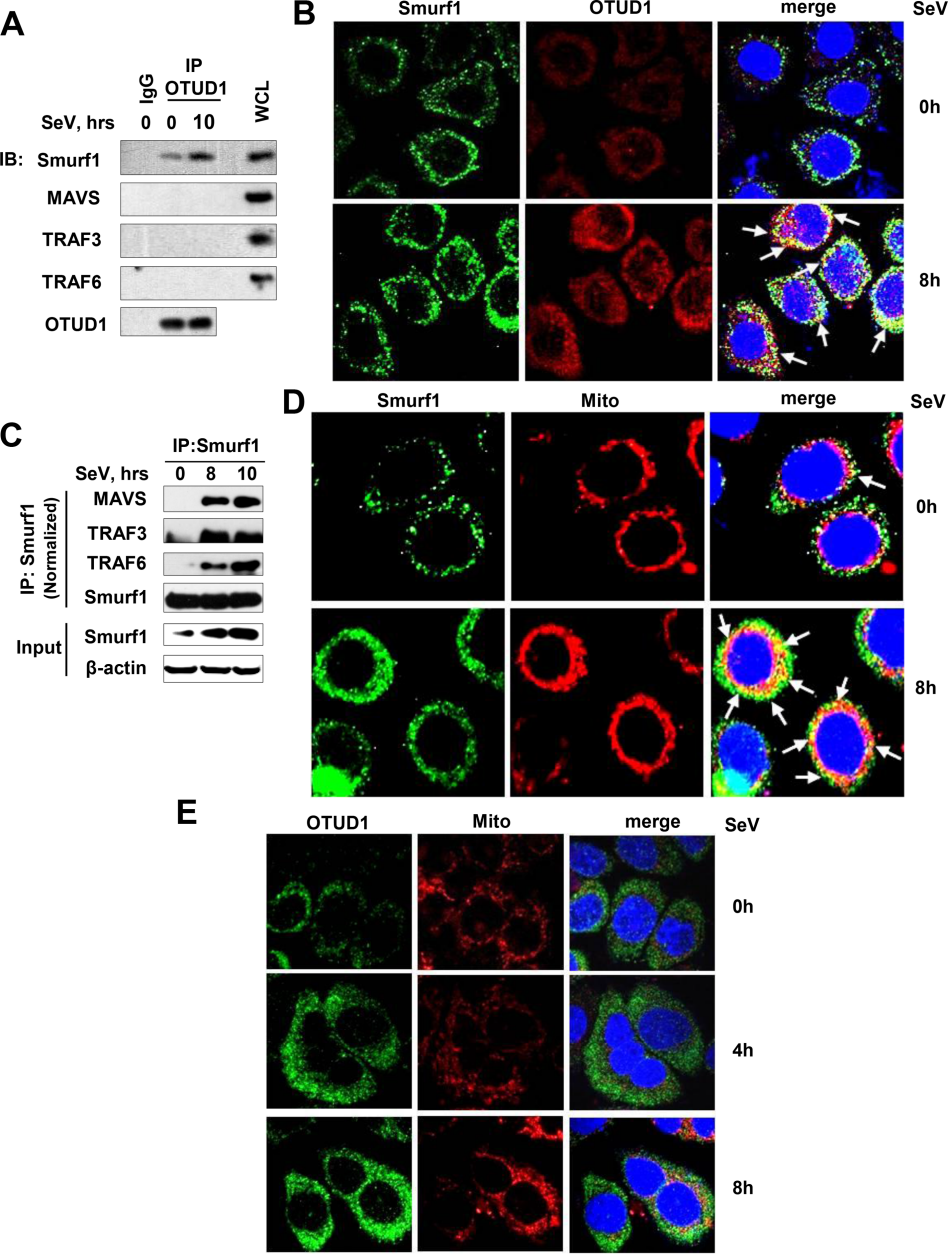
RNA virus infection promotes the interaction between OTUD1 and Smurf1, and binding of Smurf1 to MAVS/TRAF3/TRAF6. (A) 2fTGH cells were infected with SeV (MOI = 3) for 10 hr. Endogenous OTUD1 protein was immunoprecipitated, and the indicated proteins were tested by immunoblotting. The whole cell lysates (WCL) were used as a positive control. (B) HepG2 cells were infected with SeV (MOI = 3) for 8 hr, and then were stained by Smurf1 and OTUD1 antibodies. Cell nuclei were stained by DAPI. The fluorescent images were captured with the Nikon A1 confocal microscope. (C) Mouse BMDMs were infected with SeV (MOI = 3) for the indicated time. Endogenous Smurf1 protein was immunoprecipitated, and the indicated proteins were tested by immunoblotting. (D) HepG2 cells were infected as (B), and then were stained by a Smurf1 antibody. MitoTracker Red CMXRos was used for staining for mitochondria. (E) HepG2 cells were infected with SeV (MOI = 3) for 4 and 8 hr, and then were stained by an OTUD1 antibody or MitoTracker Red CMXRos. Data are representative of three independent experiments.

Given that RNA viruses activate OTUD1 expression and subsequent Smurf1 upregulation, we further analyze whether OTUD1 interacts with Smurf1, and how Smurf1 is able to affect MAVS/TRAF3/TRAF6 proteins during RNA virus infection. To this end, 2fTGH cells were infected with SeV, and then endogenous OTUD1 protein was immunoprecipitated. We found that SeV infection promoted interaction between OTUD1 and Smurf1 (Fig 7A). Similarly, by Immunofluorescence Confocal assay, we observed that SeV infection obviously promoted protein expression and accumulation of both OTUD1 and Smurf1 in cells, and enhanced co-localization between OTUD1 and Smurf1 (Fig 7B).

Next, we ask whether RNA virus infection is capable of inducing interaction between Smurf1 and MAVS/TRAF3/TRAF6. If so, where does Smurf1 interact with MAVS/TRAF3/TRAF6 in response to RNA viruses? Our results clearly showed that SeV infection significantly promoted the binding of Smurf1 to MAVS/TRAF3/TRAF6 proteins in BMDMs (Fig 7C) and 2fTGH cells (S6A Fig). Given that MAVS is a mitochondrial protein and Smurf1 can regulate MAVS/TRAF3/TRAF6 proteins during RNA virus infection, we speculate that upregulated Smurf1 proteins by viral infection could be able to localize to the mitochondria to interact with the MAVS/TRAF3/TRAF6 signalosome. Our data showed that some of Smurf1 proteins localized to the mitochondria in cells infected with SeV for 8 hr (Fig 7D), supporting the role of Smurf1 in regulating the MAVS/TRAF3/TRAF6 signalosome on the mitochondria. Interestingly, we did not observe obvious co-localization between OTUD1 and the mitochondria during SeV infection (Fig 7E), which is also consistent with the observation that OTUD1 does not interact with MAVS, TRAF3, or TRAF6 in cells infected with SeV (Fig 7A). Taken together, we think that RNA virus infection activates OTUD1 expression, which, as a trigger factor, promotes accumulation and activation of Smurf1 protein by interacting with and deubiquitinating Smurf1. Then the activated Smurf1 proteins localize to the mitochondria to interact with the MAVS/TRAF3/TRAF6 signalosome. Finally, Smurf1 promotes ubiquitination and degradation of MAVS/TRAF3/TRAF6 proteins and inhibition of IFNs production.

### RNA virus infection utilizes the OTUD1-Smurf1 axis to promote downregulation of MAVS/TRAF3/TRAF6 proteins and IFNs production

**Fig 8:**
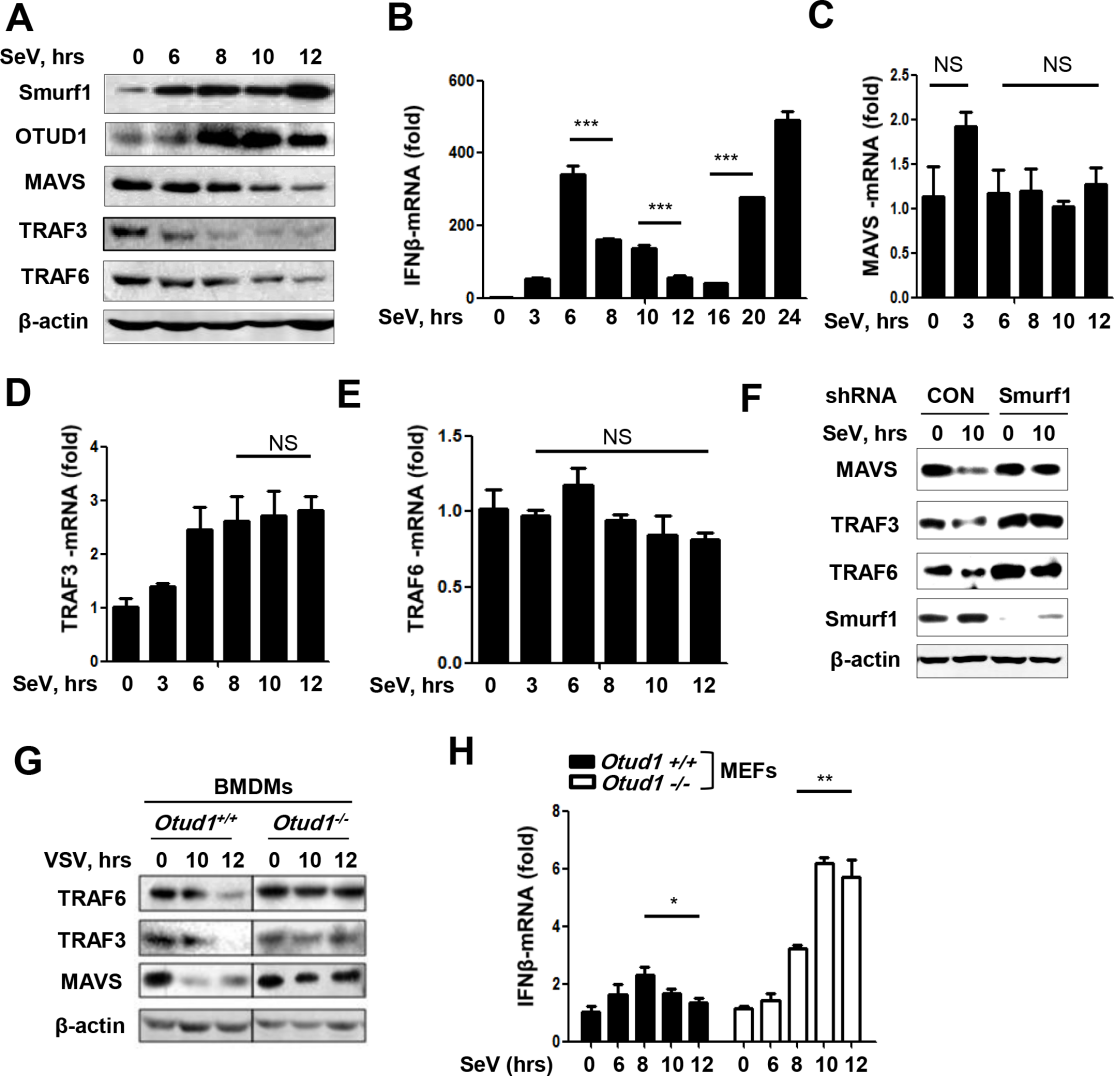
The OTUD1-Smurf1 axis regulates MAVS/TRAF3/TRAF6 levels and IFNβ induction during RNA virus infection. (A) Immunoblot analysis of the indicated proteins in the whole cell lysates of HepG2 cells infected with SeV (MOI = 3) for the indicated times. (B) 2fTGH cells were infected with SeV (MOI = 3) for the indicated times. Relative mRNA amounts of IFNβ were determined by q-PCR. The data were shown as fold change normalized to that in uninfected cells. (C-E) Relative mRNA amounts of MAVS (C), TRAF3 (D) and TRAF6 (E) in 2fTGH cells with SeV infection were determined by q-PCR. The data were shown as (B). (F) HeLa cells were transfected with shCON or shSmurf1, and then infected with SeV (MOI = 3). The levels of the indicated proteins were examined by immunoblotting. (G) BMDMs from *Otud1*^+/+^ or *Otud1*^-/-^ mice were infected with VSV (MOI = 3). The levels of the indicated proteins were examined by immunoblotting. (H) *Otud1*^+/+^ or *Otud1*^-/-^ MEF cells were infected with SeV for indicated times. Relative mRNA amounts of IFNβ were determined by q-PCR. The data were shown as fold change normalized to that in uninfected *Otud1*^+/+^ MEFs. NS, not significant (*P*>0.05); *P<0.05, **P<0.01 and ***P<0.001 (unpaired *t*-test). Error bars represent the mean and s.d., and all data are representative of three independent experiments.

Furthermore, the protein and mRNA levels of MAVS, TRAF3, and TRAF6 during RNA virus infection were observed. We noticed that some studies have shown that protein levels of MAVS, or TRAF3, or TRAF6 were downregulated to some extent after infection with different viruses [34,41–43]. Similarly, we repeatedly observed the downregulation of MAVS/TRAF3/TRAF6 protein levels within 12 hr after RNA virus infection in all tested types of cells including HepG2 (Fig 8A), HeLa (Fig 8F), and BMDMs (Fig 8G). Given that RNA virus infection downregulated MAVS/TRAF3/TRAF6 proteins, we want to know how the induction of IFNs mRNA is regulated during this stage. The results showed that the level of IFNβ mRNA was gradually reduced 8–12 hr after SeV infection (Fig 8B), which is consistent with the downregulation of the MAVS/TRAF3/TRAF6 signalosome.

The above data demonstrated that RNA virus infection downregulated MAVS/TRAF3/TRAF6 protein levels, we next questioned whether RNA virus infection downregulates mRNA levels of MAVS, TRAF3, and TRAF6. The results showed that SeV infection had no obvious effect on the levels of both MAVS and TRAF6 mRNAs within 12 hr after infection (Fig 8C and 8E). And TRAF3 mRNA levels were slightly upregulated after 6 hr infection with SeV (Fig 8D). These results suggest that RNA virus infection-induced downregulation of MAVS/TRAF3/TRAF6 proteins cannot be due to the regulation at mRNA level. In conjunction with our previous finding showing that both Smurf1 and the deubiquitinase activity of OTUD1 were required for the downregulation of MAVS/TRAF3/TRAF6 proteins (Fig 4), we first determined whether Smurf1 contributes to RNA virus-induced downregulation of MAVS/TRAF3/TRAF6 signalosome proteins. Moreover, knockdown of Smurf1 markedly inhibited SeV-induced downregulation of MAVS/TRAF3/TRAF6 proteins (Fig 8F), suggesting that Smurf1 contributes to RNA virus-induced downregulation of the MAVS/TRAF3/TRAF6 signalosome proteins.

Furthermore, we analyzed the role of OTUD1 in downregulating MAVS/TRAF3/TRAF6 proteins during RNA virus infection. Our data showed that OTUD1 deficiency in *Otud1*^*-/-*^ BMDMs blocked the downregulation of MAVS/TRAF3/TRAF6 proteins during VSV infection, when compared to *Otud1*^*+/+*^ BMDMs (Fig 8G), suggesting that OTUD1 is required for RNA virus-induced downregulation of MAVS/TRAF3/TRAF6 proteins. Moreover, the downregulation course of IFNβ mRNA within 12 hr infection with RNA viruses was blocked in *Otud1*^*-/-*^ primary liver cells (S6B Fig), as well as in *Otud1*^*-/-*^ MEFs (Fig 8H). Taken together, these data suggest that OTUD1 is responsible for downregulation of both MAVS/TRAF3/TRAF6 signalosome proteins and subsequent production of IFNβ mRNA at the early stage of RNA virus infection.

## Discussion

OTUD1 belongs to the ovarian tumor (OTU) family of the deubiquitinases [36]. Until this point, the biological functions of OTUD1 remain unknown. In this study, we uncovered that OTUD1 can downregulate protein levels of MAVS, TRAF3 and TRAF6. Importantly, OTUD1 contributes to RNA virus-induced downregulation of MAVS/TRAF3/TRAF6 proteins. As a consequence, OTUD1 inhibits MAVS/TRAF3/TRAF6 signalosome-mediated production of type I IFNs and proinflammatory cytokines during RNA virus infection (Fig 1). And *Otud1*^*-/-*^ mice produce higher levels of type I IFNs and are more resistant to RNA virus infection, when comparing to *Otud1*^*+/+*^ mice (Fig 2). Collectively, these findings reveal for the first time that OTUD1 plays critical roles in regulating host innate antiviral response.

In this study, we reveal that RNA virus infection activates expression of OTUD1, which in turn interacts with Smurf1 and upregulates Smurf1 protein levels by deubiquitination effects. We speculate that accumulation of Smurf1 proteins in cells could result in Smurf1 protein modification (such as phosphorylation, acetylation, ubiquitination, and so on), and subsequent functional activation. As observed in this study, RNA virus infection promotes Smurf1 protein accumulation in cells, and results in re-localization of Smurf1 to the mitochondria. Therefore, we can understand that RNA virus infection promotes binding of Smurf1 to MAVS/TRAF3/TRAF6 proteins, since the MAVS/TRAF3/TRAF6 signalosome is induced on the mitochondria by RNA virus infection.

Our study clearly demonstrates that the deubiquitinase OTUD1 negatively regulates RLR signaling. As a matter of fact, a great number of E3 ubiquitin ligases and deubiquitinases have been reported to be involved in the negative regulation of RLR signaling [44]. However, most of them target RIG-I or MDA5, which is the direct sensor of viral RNA components. Some E3 ubiquitin ligases including RNF125, MARCH5, Smurf2 and AIP4 can target MAVS for K48-linked ubiquitination and degradation [44]. Similarly, it has been reported that some deubiquitinases including OTUB1/2, UCHL1, MYSM1 and DUBA can remove K63-linked ubiquitination of either TRAF3 or TRAF6, thus inhibiting IFNs production during viral infection [29–33]. These reports suggest that MAVS, or TRAF3, or TRAF6 can be delicately regulated by different E3 ligases/deubiquitinases under different infection conditions (for example, different virus types, virus amounts, or different stages of infection). Here, our study uncovers a very different regulation mechanism from these E3 ubiquitin ligases and deubiquitinases. We demonstrate that at the early stage of infection, RNA virus infection rapidly activates OTUD1 expression by inducing OTUD1 mRNA in a NF-κB-dependent manner. OTUD1 upregulation results in increased protein levels and accumulation of Smurf1 by deubiquitination effects, which in turn facilitates Smurf1 localization to the mitochondria, where Smurf1 interacts with and degrades the MAVS/TRAF3/TRAF6 signalosome proteins. Finally, the deubiquitinase OTUD1 potently inhibits RLR pathway by utilizing Smurf1-MAVS/TRAF3/TRAF6 signaling at the early stage of viral infection.

Interestingly, some deubiquitinases have been implicated as positive regulators for RLR signaling. These deubiquitinases can be activated during viral infection, and then stabilize MAVS, or TRAF3, or TRAF6 protein, which finally promotes IFNs production and host antiviral defense. For example, USP25 was reported to be able to enhance the stability of TRAF3 and TRAF6, thus promoting IFNs induction by viral infection [34]. These findings could support our observation that at the late stage (around 20–24 h) of viral infection IFNs production was re-upregulated (Fig 8B), which could result from other positive regulators (for example, some deubiquitinases) of MAVS/TRAF3/TRAF6 proteins. Therefore, we think that at the late stage of viral infection, more signaling molecules take part in the regulation of the MAVS/TRAF3/TRAF6 signalosome, and finally result in the right balance of RLR-MAVS-IFNs signaling.

We clearly showed that during RNA virus infection protein levels of OTUD1 and Smurf1 significantly increased. Given that we have demonstrated that the OTUD1-Smurf1 axis promoted downregulation of MAVS/TRAF3/TRAF6 proteins, we think that the levels of MAVS, TRAF3 and TRAF6 should be decreased at certain time points during RNA virus infection. However, until this point there are few studies that clearly analyzed the dynamics of downregulation of MAVS/TRAF3/TRAF6 proteins during viral infection. It could be partially because most of previous studies focused on the signaling which regulates only one component of this MAVS/TRAF3/TRAF6 signalosome. Here, our findings uncover the phenomenon of MAVS/TRAF3/TRAF6 downregulation at the early stage of viral infection, and partially clarify the mechanisms of the dynamics of downregulation of the signalosome and IFNs induction.

In summary, we elucidate that RNA virus infection upregulates *Otud1* expression via the NF-κB signaling pathway. RNA virus-induced OTUD1 protein regulates Smurf1 ubiquitination and increases the level of Smurf1 protein. RNA virus infection also promotes the binding of Smurf1 to MAVS/TRAF3/TRAF6 proteins, thus downregulating MAVS/TRAF3/TRAF6 protein levels by ubiquitination and proteasome-dependent degradation. Consequently, OTUD1 negatively regulates RNA virus-triggered production of type I IFNs and proinflammatory cytokines. This model explains why OTUD1 deficiency protects mice from RNA virus infection. Our findings have revealed a novel negative feedback regulation of innate antiviral immune response, and could provide potential therapeutic targets for viral infection-related diseases.

## Materials and methods

### Mice

*Otud1*^*-/-*^ mice on a C57BL/6 background were generated by Cyagen Biosciences Inc. (Guangzhou, Guangdong, China). Mice were maintained and bred in special-pathogen-free (SPF) conditions in the Experimental Animal Center of Soochow University. All knockout mice were identified via PCR of genomic DNA from tails using the primers: 5’-CTGTGGCGCAGCA CGAATTGGGT-3’ (Forward), 5’-ATGTGCGCCGTGGACGTGAAGT-3’ (Reverse). 6–8 weeks old mice were used in most of experiments.

### Ethics statement

Animal care and use protocol adhered to the National Regulations for the Administration of Affairs Concerning Experimental Animals. All protocols and procedures for mice study were performed in accordance with the Laboratory Animal Management Regulations with approval of the Scientific Investigation Board of Soochow University. The project license number is 201705A299.

### Cells isolation from mice

Bone marrows were prepared from the 8 weeks adult mice. And the cells were cultured in RPMI medium supplemented with GM-CSF (50 ng/ml) for 7 days for BMDMs differentiation. For the MEFs, *Otud1*^*+/+*^ or *Otud1*^*-/-*^ embryos were obtained in the pregnant 13 days mice. The primary mouse liver cells were isolated from mouse liver tissues, which were cut into pieces and digested by erythrocyte lysis buffers. After centrifugation, cells were collected and cultured in PRIM medium and prepared for further experiments.

### Viral infection in vitro

Vesicular Stomatitis Virus (VSV) and Sendai virus (SeV) were provided by Dr. Chen Wang (Shanghai Institutes for Biological Science, Chinese Academy of Science, China). Influenza A Virus (H1N1, PR/8/34) was from Dr. Jianfeng Dai (Institutes of Biology and Medical Sciences, Soochow University). Herpes simplex virus (HSV) was from Dr. Chunfu Zheng (Soochow University). Cells were prepared for viral infection to detect gene production and signaling expression. Cells in the serum-free medium were infected with VSV, SeV, H1N1 or HSV for 1.5 hrs. Then the supernatant was removed and cells were returned to the medium containing 10% FBS for the indicated times.

### VSV infection in vivo

Eight weeks old mice and VSV were prepared for the in vivo viral infection by intraperitoneal injection or intranasal injection as described. The expression of IFNβ, IL-6, TNFα and VSVG were detected in the organs via q-PCR. 3 days of VSV-infected mice were collected for immunohistochemistry staining. Survival observation continued until 15 days.

### TCID50 assay

Viral titers were determined by the 50% tissue culture infectious dose (TCID50) and standard curves were presented as described [45]. Briefly, *Otud1*^+/+^ or *Otud1*^-/-^ MEFs were infected with either VSV or HSV for 10 hr. Cultural supernatants containing the viruses were diluted with DMEM serially, and then were put on the monolayer of Vero cells in 96-well plates with 8 repetitions. TCID50 was measured for viral titers after 3 days.

### Immunohistochemistry assay

Lungs, livers and kidneys dissected from 3 day-infected mice were fixed in 4% formaldehyde solution and embedded into paraffin. Paraffin sections were placed on a slide and incubated with a VSVG antibody. The slides were then stained with hematoxylin-eosin solution. The histological changes were finally examined by the positive fluorescence microscopy.

### Immunofluorescence microscopy

Immunofluorescence microscopy was performed as described previously [35]. Briefly, cells were infected with SeV for 4 hr or 8 hr, and then incubated with MitoTracker Red CMXRos (40741ES50, Yeasen) for 30 min. Cells were permeabilized with 0.5% Triton X-100 and blocked with 5% BSA, and then were incubated with either an anti-OTUD1 antibody (Abcam, ab122481) or an anti-Smurf1 (Santa Cruz, sc-100616) antibody overnight, followed by staining with either 488 goat anti-mouse IgG (Alexa Fluor, A11001) or 594 goat anti-rabbit IgG (Alexa Fluor, A11012). Cell nuclei were stained with DAPI for 30 min, and the fluorescent images were captured with the Nikon A1 confocal microscope.

### Analysis of serum ELISA

IFNβ ELISA Kits (Elabscience, E-EL-M0033C) were used to test the concentrations of IFNβ protein in sera from VSV-infected mice for 24 hr.

### Tissue RNA extraction

Tissues were extracted by the homogenizer from the infected mice. After the red blood cells were broken, tissue cells were cleaved with the Trizol reagent. Total RNAs were extracted according to the manufacturer's instructions (Invitrogen).

### Cell culture, transfection and reagents

HEK293T, HeLa, HT1080, 2fTGH and HepG2 cells were obtained from ATCC. U3A cells were provided by Dr. Guoqiang Chen (Shanghai Jiao Tong University). Vero cells were gifts from Dr. Chunfu Zheng (Soochow University). All cells were cultured at 37°C under 5% CO2 in Dulbecco’s modified Eagle’s medium (DMEM; HyClone) supplemented with 10% FBS (GIBCO, Life Technologies), 100 units/ml penicillin, and 100 μg/ml streptomycin.

For transfection, LongTrans (UCallM, TF/07), PEI (Polyetherimide) and Lipofectamine 3000 (Invitrogen, L3000-015) were used. The plasmids and reagents required in the experiment were as following: Flag-OTUD1 plasmids were obtained from Dr. J Wade Harper (Harvard Medical School, Addgene plasmids). IFNβ (P125)-Luc, ISRE-Luc, Renilla, HA-Ub and mutant plasmids (HA-R48K, HA-R63K) were gifts from Dr. Serge Y. Fuchs (University of Pennsylvania). Flag-Smurf1, Flag-TRAF3, Flag-TRAF6 and Flag-MAVS were provided by Dr. Chengjiang Gao (Shandong University, China). Flag-OTUD1 (CH) was generated by QuickChange site-Directed Mutagenesis Kit (Stratagene). The reagents are as following: MG132 (Sigma, C2211), cGAMP (InvivoGen, tlrl-nacga23), Poly(I:C) (HMW) Rhodamine (InvivoGen, tlrl-picr), LPS (Sigma, L2630). ISD was a gift from Dr. Chunfu Zheng (Soochow University, China).

### Q-PCR assay

Isolation of mRNAs from cells was performed by Trizol, and then reverse transcription kits (Thermo, K1622) were used for cDNA synthesis. The change-in-cycling-threshold (2^-ΔΔCt^) method was utilized for calculation of the relative gene expression levels. Quantification of all target genes was normalized to the control gene *β-actin*, and all data are shown as fold change normalized to that in either unstimulated or uninfected cells accordingly. The primers used in qPCR were listed: *Otud1* forward, 5'-GGGGAGTTTATCATCGCTGCT-3' and reverse, 5'-TGAGCCAACTGAGCCAAATAC-3'; *Smurf1* forward, 5'-ATTCGAT AACCATTAGCGTGTGG-3' and reverse, 5'-CGCCGGTTCCTATTCTGTCTC-3'; *Traf3* forward, 5'-ATGCTGAGTGTGCACGACAT-3' and reverse, 5'-TAGAC CCTGGCACATCTTA-3'; *Traf6* forward, 5'-GCACAAGATGGAACTGAGACA-3' and reverse, 5'-TGACATTTGCCAAAGGACAG-3'; *Mavs* forward, 5'-TAAAC AGGGTGCAGAGAGTGA-3' and reverse, 5'-GATTGGTGAGCGCATTAGAA-3'; *SeV* forward, 5'-GATGACGATGCCGCAGCAGTAG-3' and reverse, 5'-CCTC CGATGTCAGTTGGTTCACTC-3'; *Vsvg* forward,5'-ACGGCGTACTTCCAGAT GG-3' and reverse, 5'-CTCGGTTC AAGATCCAGGT-3'; H1N1 forward, 5'-TTC TAACCGAGGTCGAAACG-3' and reverse, 5'-ACAAAGCGTCTACGCTG CAG-3'; *Ifit1* forward,5'-GCCTATCGCCAAGATTTAGATGA-3' and reverse, 5'-TTCTGGATTTAACCGGACAGC-3'; *Isg15* forward,5'-GGTGTCCGTGACT AACTCCAT-3'and reverse, 5'-CTGTACCACTAGCATCACTGTG-3'; *Isg20* forward, 5'-GAAGCGAAGGTTCTTGGAACA-3' and reverse, 5'-GCCATCTAC TCTTGAAGTTTCCC-3'; *Isg54* forward,5'-GGAGAGCAATCTGCGACAG-3' and reverse, 5'-GCTGCCTCATTTAGACCTCTG-3'; *Human-IFNβ* forward, 5'-CATT ACCTGAAGGCCAAGGA-3' and reverse, 5'-CAGCATCTGCTGGTTGAAGA- 3'; *Mouse-IFNβ* forward, 5'-CTTCGTGTTTGGTAGTGATGGT-3' and reverse, 5'-GGGGATGATTTCCAGCCGA-3'; *Il-6* forward, 5'-GGCGGATCGGATGTT GTGAT-3' and reverse, 5'-GGACCCCAGACAATCGGTTG-3' *Actb* forward, 5'-ACCAACTGGGACGACATGGAGAAA-3' and reverse, 5'-ATAGCACAGCCT GGATAGCAACG-3'.

### Assay of luciferase activity

Cells were transfected with specific plasmids together with ISRE-Luc and Renilla plasmids. Cells were infected with viruses before harvested. P125-Luc plasmid was used in the IFNβ promoter assay. The luciferase activity was tested by using the Dual Luciferase Reporter Assay System (Promega, E1910). Three independent experiments were performed and were shown as the average mean ± standard derivation (s.d.).

### Immunoprecipitation and immunoblotting

Immunoprecipitation and immunoblotting were carried out as described previously [46]. The following antibodies were used: IRF3 (1:1000, Santa Cruz, sc-9082), p-IRF3 (1:1000, Cell Signaling, 4947S), OTUD1 (1:1000, Abcam, ab182511), Flag (1:5000, Sigma, F7425), β-actin (1:5000, Proteintech, 66009-1-Ig), Myc (1:5000, Abmart, m2002), MAVS (1:1000, Santa Cruz, sc-166583), TRAF3 (1:1000, Cell Signaling, 4729S), TRAF6 (1:1000, Abcam, ab94720), TBK1 (1:1000, Cell Signaling, 3013S), RIG-I (1:1000, Cell Signaling, 4200S), Smurf1 (1:1000, Santa Cruz, sc-100616), Smurf2 (1:1000, Santa Cruz, sc-25511), Ub (1:1000, Santa Cruz, sc-8017), HA (1:5000, Abcam, ab9110), K48-Ub (1:1000, Cell Signaling, 4289S), and VSVG (1:5,000, Santa Cruz, sc-66180).

### Statistical analysis

Statistical significance between groups was performed using two-tailed Student's t-test or Gehan-Breslow-Wilcoxon test. Densitometry quantification was made with ImageJ software. P values less than 0.05 were considered significant. *p < 0.05, **p < 0.01, ***p < 0.001; NS, not significant. Kaplan-Meier survival curves were generated and analyzed for mouse survival study performed in Graph Pad Prism 5.0.

## Supporting information

S1 Fig OTUD1 negatively regulates interferon signaling. (A) HEK293T cells were transfected with control shRNAs (shCON) or OTUD1 shRNAs (shOTUD1) for 72 hr. OTUD1 protein in the whole cell lysates was detected by immunoblotting as indicated. (B and C) HEK293T cells were transfected with either shOTUD1 (B) or Flag-OTUD1 (C), together with ISRE-Luc and Renilla plasmids. The luciferase activity was measured and shown as fold change normalized to that of uninfected cells in the shCON group. (D) *Otud1*^+/+^ or *Otud1*^-/-^ MEFs were infected with H1N1. 10 hr after infection, IFNβ mRNAs were analyzed by quantitative real time PCR (q-PCR). The data were shown as fold change normalized to that in uninfected *Otud1*^+/+^ MEFs. (E) *Otud1*^+/+^ or *Otud1*^-/-^ MEFs were transfected with Poly(I:C) (2 μg/ml) (left) or were stimulated with LPS (2 μg/ml) (right). After 10 hr, the IFNβ mRNAs were analyzed by q-PCR. The data were shown as fold change normalized to that in unstimulated *Otud1*^+/+^ MEFs. (F) *Otud1*^+/+^ or *Otud1*^-/-^ mice were infected with VSV (2X10^7^ pfu per mouse) for 24 hr. The production of serum IFN-β protein was detected by ELISA. The data were shown as (D). (G) HEK293T cells were transfected with shCON or shOTUD1. The levels of ISG15, ISG20 and ISG54 mRNA were determined by q-PCR analysis after 10 hr infection with SeV. The data were shown as (B). (H, I) *Otud1*^+/+^ or *Otud1*^-/-^ primary liver cells were infected with SeV for 10 hr. The relative mRNA amounts of IL-6 (H) and TNFα (I) were determined by q-PCR. The data were shown as (D).*P<0.05, **P<0.01 and ***P<0.001 (unpaired t-test). Error bars represent the mean and s.d. of three independent experiments.
(TIF)

S2 Fig OTUD1 downregulates IFN-β luciferase activity. (A) Relative IFNβ luciferase activity in HEK293T cells transfected with shRNAs plasmids (shCON or shOTUD1), together with IFNβ promoter (P125)-Luc and Renilla, relative IFNβ luciferase was measured after SeV infection 10 hr. The data were shown as fold change normalized to that of uninfected cells in the control group. (B) HEK293T cells were transfected with vector plasmids or Flag-OTUD1, together with P125-Luc and Renilla as shown. 10 hr after SeV infection, the luciferase activity was measured as indicated. The data were shown as (A). (C) Immunoblot analysis of phosphorylated IRF3 (p-IRF3) in HeLa cells transfected with expression plasmids (vector or Flag-OTUD1). (D) Immunoblot analysis of dimeric IRF3 via native-PAGE in HeLa cells transfected with vector or Flag-OTUD1 and then infected with or without SeV for 12 hr. *P<0.05, **P<0.01 and ***P<0.001 (unpaired t-test). Data are representative of three independent experiments.
(TIF)

S3 Fig Myc-OTUD1 interacts with Flag-MAVS, Flag-TRAF3 and Flag-TRAF6. (A-C) HEK293T cells were transfected with Myc-OTUD1, together with Flag-MAVS (A), or Flag-TRAF3 (B), or Flag-TRAF6 (C). Immunoprecipitation and immunoblotting were carried out as indicated. Data are representative of three independent experiments.
(TIF)

S4 Fig OTUD1 regulates protein levels of MAVS, TRAF3, TRAF6 and Smurf1. (A-C) HEK293T cells were transfected with Myc-OTUD1, together with Flag-MAVS (A), or Flag-TRAF3 (B), or Flag-TRAF6 (C). Immunoblotting was performed as indicated. (D) HEK293T cells were transfected with increasing amounts of Myc-OTUD1. The levels of Smurf1 and Smurf2 were analyzed by immunoblotting. (E) HEK293T cells were transfected with Myc-OTUD1 and HA-Ub-R48K (K48-only), together with Flag-MAVS, or Flag-TRAF3, or Flag-TRAF6 as indicated. Immunoprecipitation and immunoblot analysis were carried out as indicated. (F) HEK293T cells were treated with MG132 (20 μM) for 0, 8, 12 hr. The levels of OTUD1 and MAVS were detected by immunoblotting as indicated. Data are representative of three independent experiments.
(TIF)

S5 Fig The expression of OTUD1 and Smurf1 in response to viruses. (A) Q-PCR analysis of OTUD1 mRNA expression in 2fTGH cells infected with VSV (MOI = 3) for 0, 3, 6 hr. The data were shown as fold change normalized to that in uninfected cells. (B) Q-PCR analysis of OTUD1 mRNA expression in MEFs cells infected with HSV (MOI = 3) for 0, 3, 6 hr. The data were shown as (A). (C) MEFs were transfected with Poly(I:C) (2 μg/ml). After 10 hr, the OTUD1 mRNAs were analyzed by q-PCR. The data were shown as fold change normalized to that in unstimulated cells. (D and E) Q-PCR analysis of OTUD1 mRNA expression in MEFs stimulated by ISD (2 μg/ml) (D) or by cGAMP (1 μg/ml) (E) for 10 hr. The data were shown as fold change normalized to that in unstimulated cells. (F) Immunoblot analysis of Smurf1 protein in the whole cell lysates from HeLa cells infected with SeV (MOI = 3) for the indicated times. **P<0.01, NS, not significant (*P*>0.05) (unpaired t-test). Error bars represent the mean and s.d., and all data are representative of three independent experiments.
(TIF)

S6 Fig RNA virus infection promotes the interaction between Smurf1 of MAVS/TRAF3/TRAF6 proteins, and OTUD1 deficiency regulates RNA virus-induced IFNβ production. (A) 2fTGH cells were infected with SeV as indicated. Endogenous Smurf1 protein was immunoprecipitated, followed by immunoblot analysis of the indicated proteins. (B) The primary liver cells from *Otud1*^*+/+*^ or *Otud1*^*-/-*^ mice were infected with SeV (MOI = 3). Relative amounts of IFNβ mRNA were determined by q-PCR. The data were shown as fold change normalized to that in uninfected *Otud1*^*+/+*^ cells. *P<0.05, NS, not significant (unpaired t-test). Error bars represent the mean and s.d. Data are representative of three independent experiments.
(TIF)
