# Supplementary figures and images for: Induction of OTUD1 by RNA viruses potently inhibits innate immune responses by promoting degradation of the MAVS/TRAF3/TRAF6 signalosome
Source: PLoS Pathog. 2018 May 7;14(5):e1007067. doi: 10.1371/journal.ppat.1007067 (PMC5957451; doi:10.1371/journal.ppat.1007067)

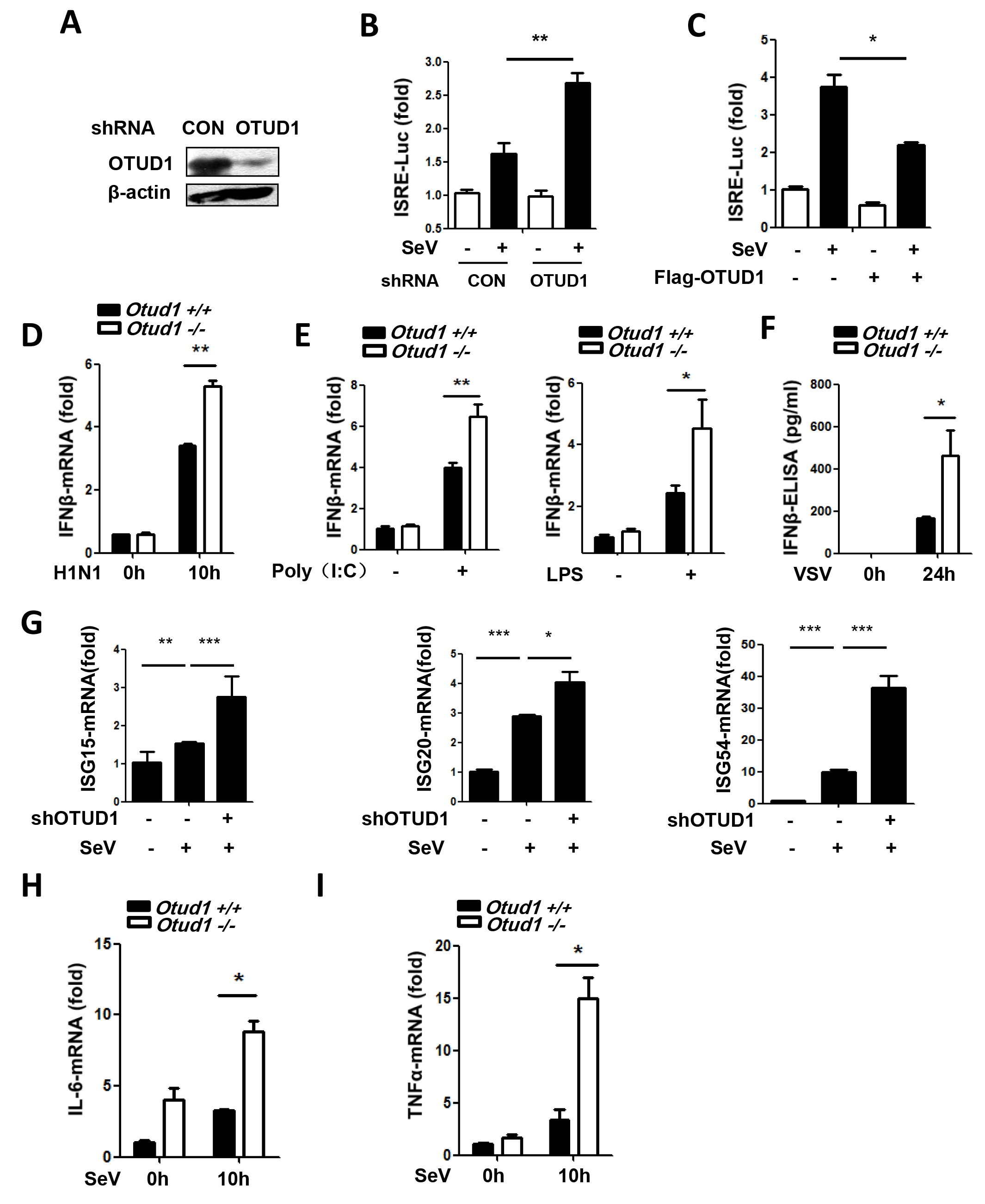

Supplement: S1 Fig — (A) HEK293T cells were transfected with control shRNAs (shCON) or OTUD1 shRNAs (shOTUD1) for 72 hr. OTUD1 protein in the whole cell lysates was detected by immunoblotting as indicated. (B and C) HEK293T cells were transfected with either shOTUD1 (B) or Flag-OTUD1 (C), together with ISRE-Luc and Renilla plasmids. The luciferase activity was measured and shown as fold change normalized to that of uninfected cells in the shCON group. (D) Otud1+/+ or Otud1-/- MEFs were infected with H1N1. 10 hr after infection, IFNβ mRNAs were analyzed by quantitative real time PCR (q-PCR). The data were shown as fold change normalized to that in uninfected Otud1+/+ MEFs. (E) Otud1+/+ or Otud1-/- MEFs were transfected with Poly(I:C) (2 μg/ml) (left) or were stimulated with LPS (2 μg/ml) (right). After 10 hr, the IFNβ mRNAs were analyzed by q-PCR. The data were shown as fold change normalized to that in unstimulated Otud1+/+ MEFs. (F) Otud1+/+ or Otud1-/- mice were infected with VSV (2X107 pfu per mouse) for 24 hr. The production of serum IFN-β protein was detected by ELISA. The data were shown as (D). (G) HEK293T cells were transfected with shCON or shOTUD1. The levels of ISG15, ISG20 and ISG54 mRNA were determined by q-PCR analysis after 10 hr infection with SeV. The data were shown as (B). (H, I) Otud1+/+ or Otud1-/- primary liver cells were infected with SeV for 10 hr. The relative mRNA amounts of IL-6 (H) and TNFα (I) were determined by q-PCR. The data were shown as (D).*P<0.05, **P<0.01 and ***P<0.001 (unpaired t-test). Error bars represent the mean and s.d. of three independent experiments. (TIF) [file ppat.1007067.s001.tif]

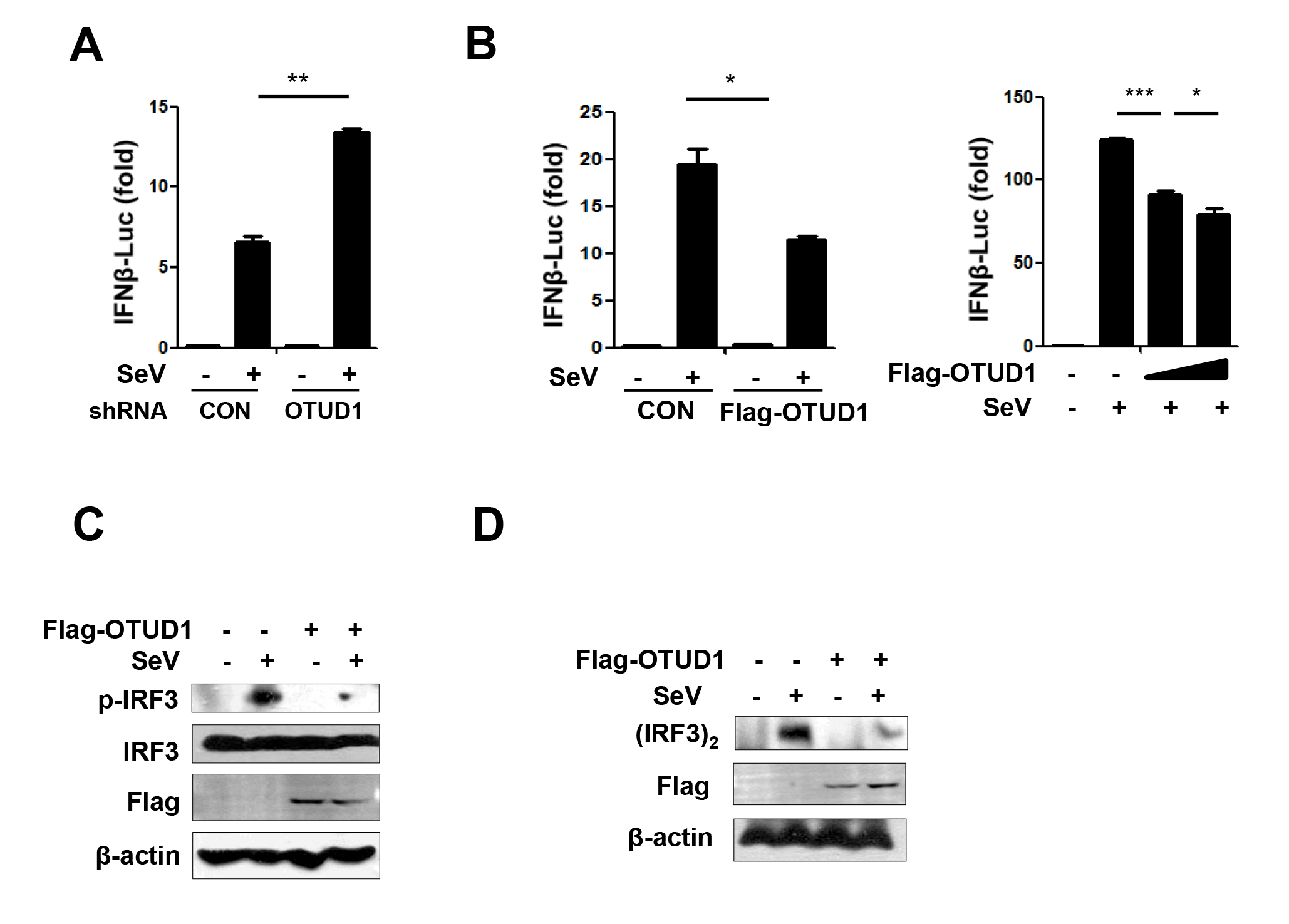

Supplement: S2 Fig — (A) Relative IFNβ luciferase activity in HEK293T cells transfected with shRNAs plasmids (shCON or shOTUD1), together with IFNβ promoter (P125)-Luc and Renilla, relative IFNβ luciferase was measured after SeV infection 10 hr. The data were shown as fold change normalized to that of uninfected cells in the control group. (B) HEK293T cells were transfected with vector plasmids or Flag-OTUD1, together with P125-Luc and Renilla as shown. 10 hr after SeV infection, the luciferase activity was measured as indicated. The data were shown as (A). (C) Immunoblot analysis of phosphorylated IRF3 (p-IRF3) in HeLa cells transfected with expression plasmids (vector or Flag-OTUD1). (D) Immunoblot analysis of dimeric IRF3 via native-PAGE in HeLa cells transfected with vector or Flag-OTUD1 and then infected with or without SeV for 12 hr. *P<0.05, **P<0.01 and ***P<0.001 (unpaired t-test). Data are representative of three independent experiments. (TIF) [file ppat.1007067.s002.tif]

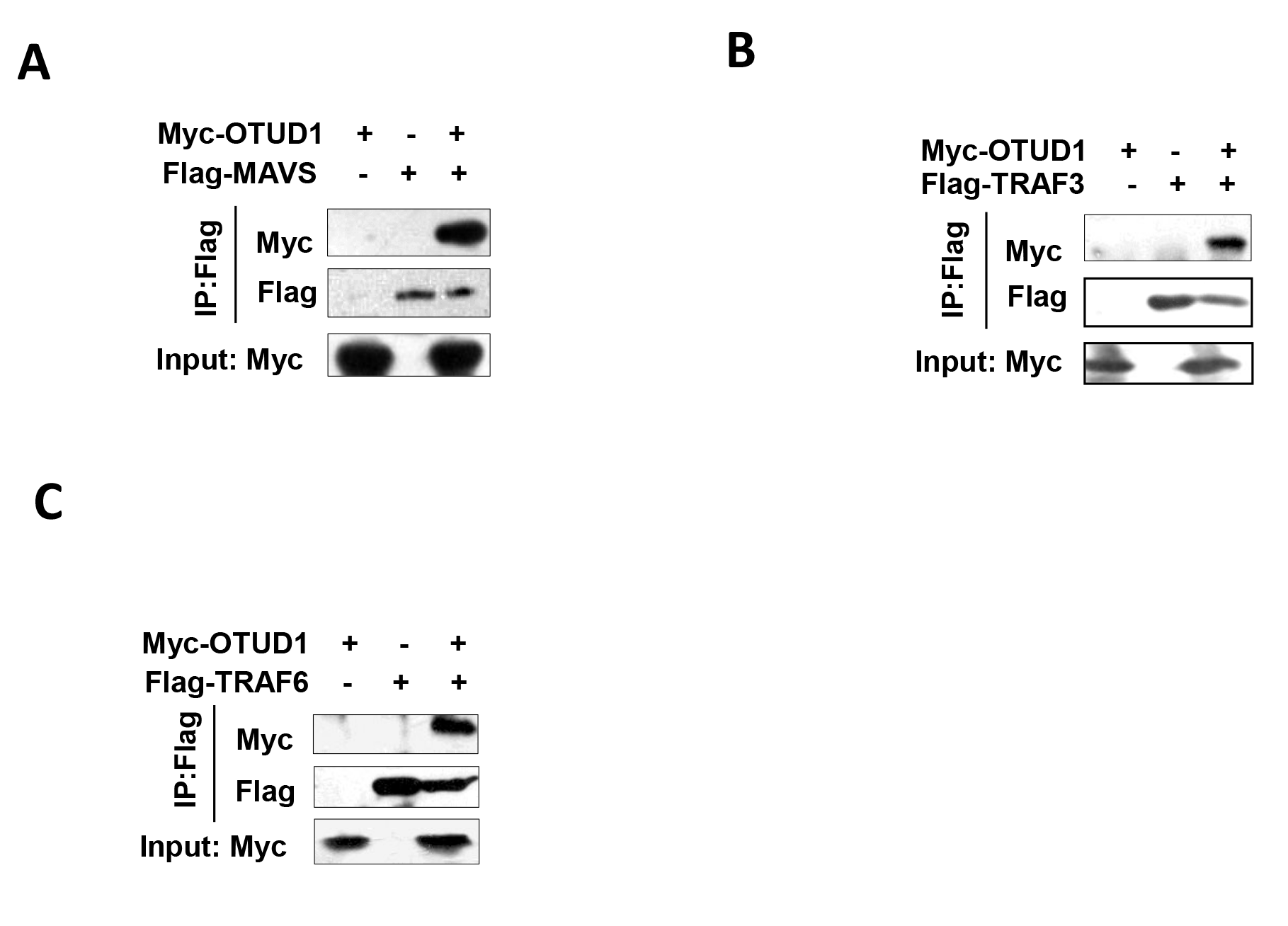

Supplement: S3 Fig — (A-C) HEK293T cells were transfected with Myc-OTUD1, together with Flag-MAVS (A), or Flag-TRAF3 (B), or Flag-TRAF6 (C). Immunoprecipitation and immunoblotting were carried out as indicated. Data are representative of three independent experiments. (TIF) [file ppat.1007067.s003.tif]

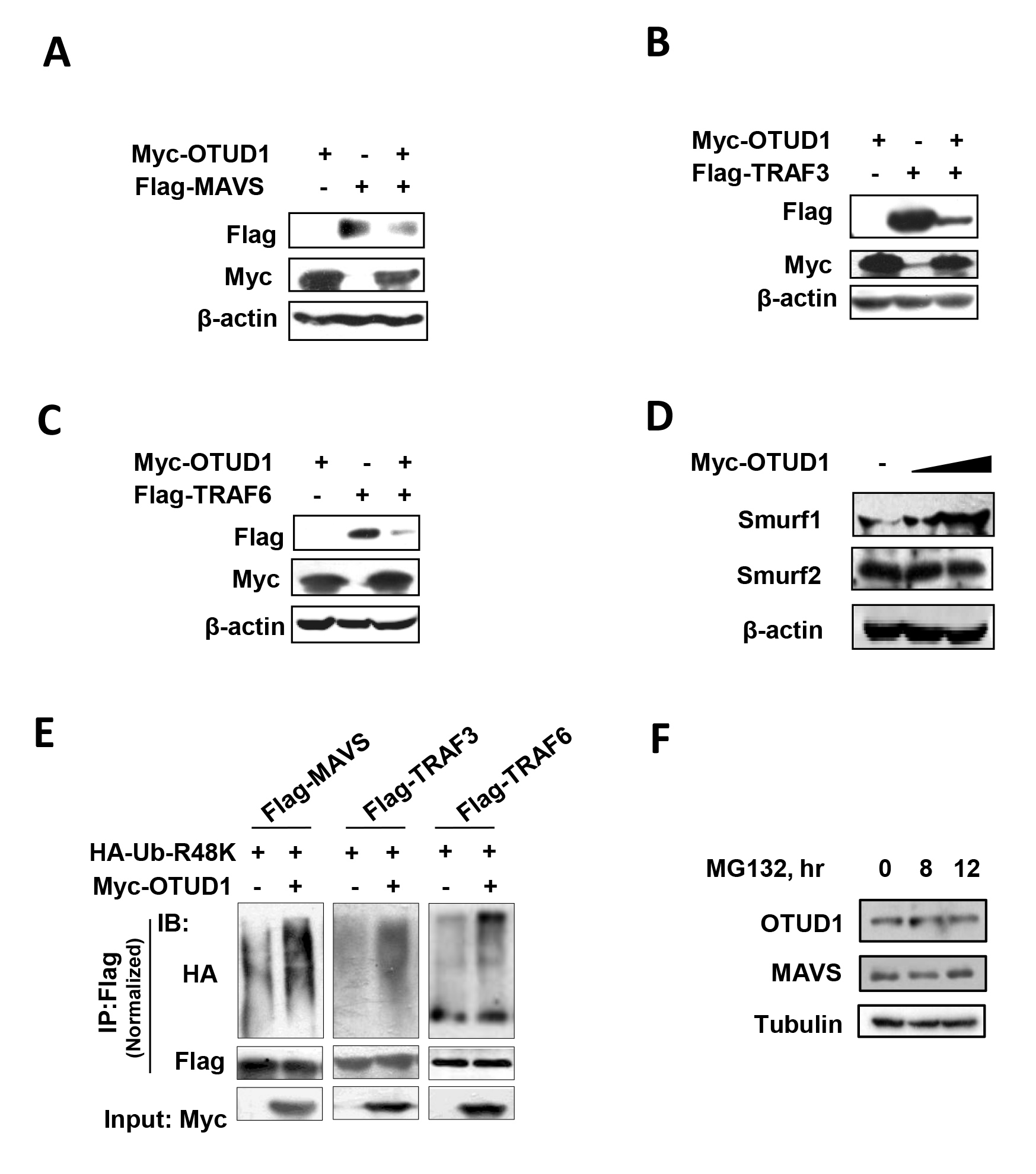

Supplement: S4 Fig — (A-C) HEK293T cells were transfected with Myc-OTUD1, together with Flag-MAVS (A), or Flag-TRAF3 (B), or Flag-TRAF6 (C). Immunoblotting was performed as indicated. (D) HEK293T cells were transfected with increasing amounts of Myc-OTUD1. The levels of Smurf1 and Smurf2 were analyzed by immunoblotting. (E) HEK293T cells were transfected with Myc-OTUD1 and HA-Ub-R48K (K48-only), together with Flag-MAVS, or Flag-TRAF3, or Flag-TRAF6 as indicated. Immunoprecipitation and immunoblot analysis were carried out as indicated. (F) HEK293T cells were treated with MG132 (20 μM) for 0, 8, 12 hr. The levels of OTUD1 and MAVS were detected by immunoblotting as indicated. Data are representative of three independent experiments. (TIF) [file ppat.1007067.s004.tif]

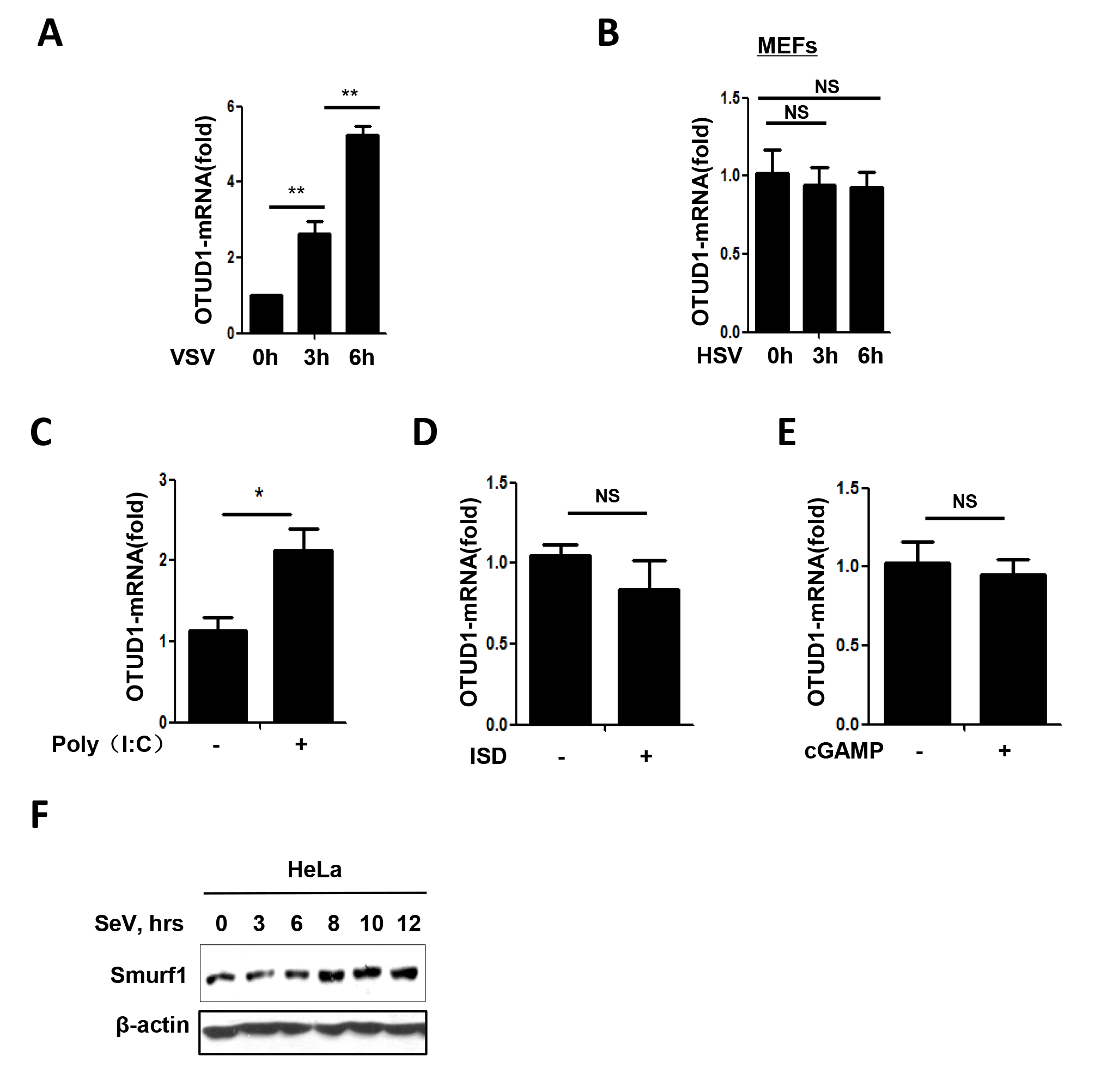

Supplement: S5 Fig — (A) Q-PCR analysis of OTUD1 mRNA expression in 2fTGH cells infected with VSV (MOI = 3) for 0, 3, 6 hr. The data were shown as fold change normalized to that in uninfected cells. (B) Q-PCR analysis of OTUD1 mRNA expression in MEFs cells infected with HSV (MOI = 3) for 0, 3, 6 hr. The data were shown as (A). (C) MEFs were transfected with Poly(I:C) (2 μg/ml). After 10 hr, the OTUD1 mRNAs were analyzed by q-PCR. The data were shown as fold change normalized to that in unstimulated cells. (D and E) Q-PCR analysis of OTUD1 mRNA expression in MEFs stimulated by ISD (2 μg/ml) (D) or by cGAMP (1 μg/ml) (E) for 10 hr. The data were shown as fold change normalized to that in unstimulated cells. (F) Immunoblot analysis of Smurf1 protein in the whole cell lysates from HeLa cells infected with SeV (MOI = 3) for the indicated times. **P<0.01, NS, not significant (P>0.05) (unpaired t-test). Error bars represent the mean and s.d., and all data are representative of three independent experiments. (TIF) [file ppat.1007067.s005.tif]

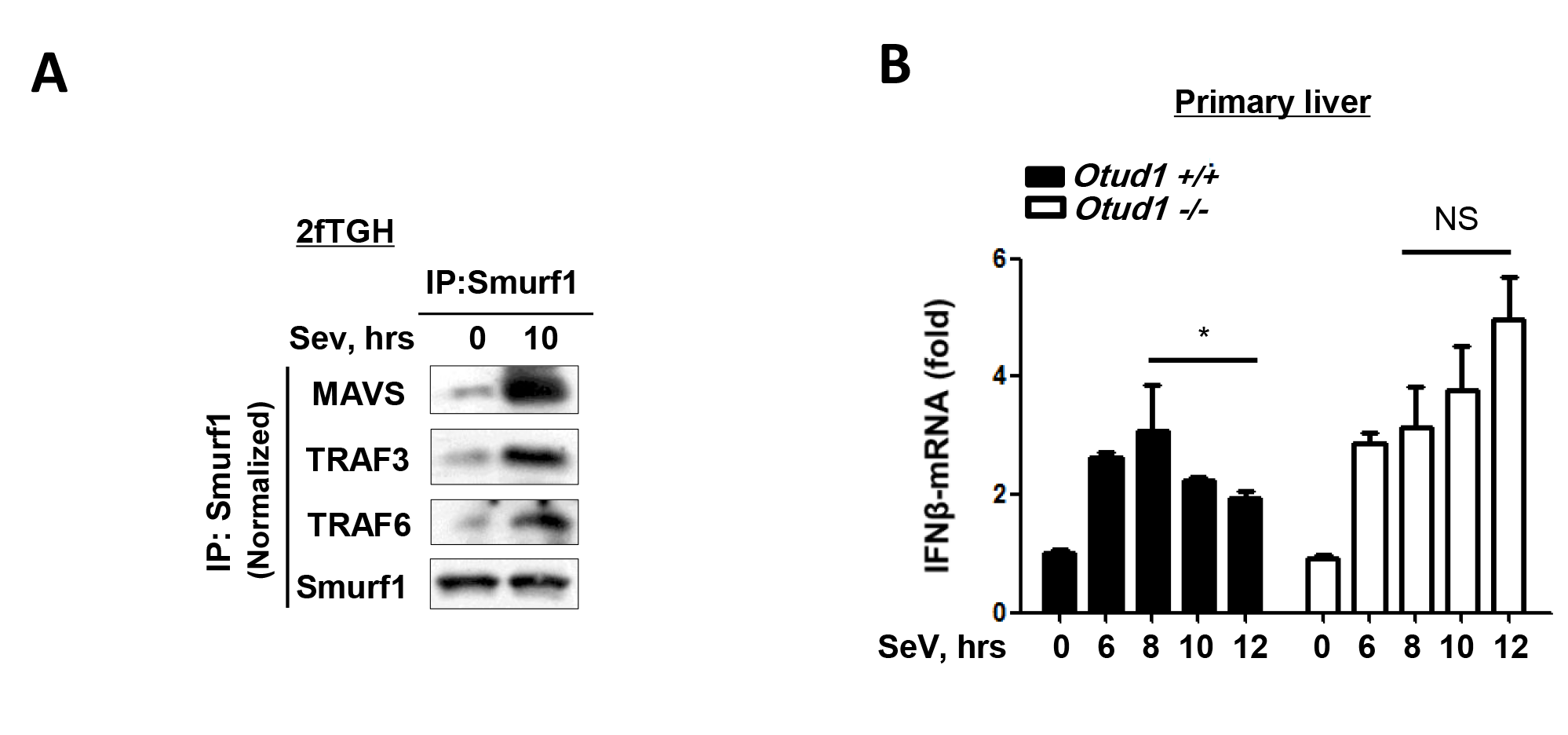

Supplement: S6 Fig — (A) 2fTGH cells were infected with SeV as indicated. Endogenous Smurf1 protein was immunoprecipitated, followed by immunoblot analysis of the indicated proteins. (B) The primary liver cells from Otud1+/+ or Otud1-/- mice were infected with SeV (MOI = 3). Relative amounts of IFNβ mRNA were determined by q-PCR. The data were shown as fold change normalized to that in uninfected Otud1+/+ cells. *P<0.05, NS, not significant (unpaired t-test). Error bars represent the mean and s.d. Data are representative of three independent experiments. (TIF) [file ppat.1007067.s006.tif]
